# Strain-Tunable Electronic and Optical Properties of KSnI_3_ Perovskite Polymorphs: From Structural Stability to Optoelectronic Potential

**DOI:** 10.1021/acsomega.6c00784

**Published:** 2026-05-02

**Authors:** Aynur Ayvalik, Kadir Can Dogan, Zebih Cetin, Mehmet Yagmurcukardes, Humeyra Orucu

**Affiliations:** † Department of Material Science and Engineering, The Graduate School of Natural and Applied Sciences, 37509 Ege University, 35100 Izmir, Turkey; ‡ Department of Physics, 52972 Izmir Institute of Technology, 35430 Izmir, Turkey; § Department of Photonics, Izmir Institute of Technology, 35430 Izmir, Turkey; ∥ Department of Physics, Ege University, 35100 Izmir, Turkey

## Abstract

The interplay between crystal symmetry and strain provides a powerful, yet underexplored, route for tailoring the optoelectronic properties of lead-free KSnI_3_ perovskites. In the present work, a comprehensive structure–property-strain framework is established through systematic first-principles analysis, revealing how symmetry and mechanical deformation govern the electronic structure and optical response across multiple polymorphs. Only the orthorhombic (*Pnma-1* and *Pnma-2*) and monoclinic (*P2*_1_/*m*) phases are found to be dynamically, thermally, and mechanically stable, whereas the tetragonal (*P4/mmm* and *P4/mbm*) phases exhibit lattice instabilities. Electronically, *Pnma-1* and *P2*_1_/*m* exhibit indirect semiconducting behavior, while *Pnma-2*, *P4/mmm*, and *P4/mbm* possess direct band gaps, enabling a symmetry-driven optoelectronic functionality. All stable phases exhibit strong polarization-dependent visible-UV absorption with *Pnma-2* extending into the infrared, yielding a broadband optical response. Notably, the *P2*_1_/*m* phase is distinguished by a y-polarized absorption coefficient of 1.06 × 10^8^ cm^–1^ at 1.88 eV and a reflectivity of up to 82%, highlighting its potential for high-performance optical coating applications. Going beyond static properties, biaxial and triaxial strain emerge as efficient routes for tuning the band gap and optical response of KSnI_3_ phases over a wide energy range. Overall, our findings demonstrate KSnI_3_ as a highly tunable lead-free perovskite platform where symmetry and strain serve as key design parameters for optoelectronic applications.

## Graphical abstract

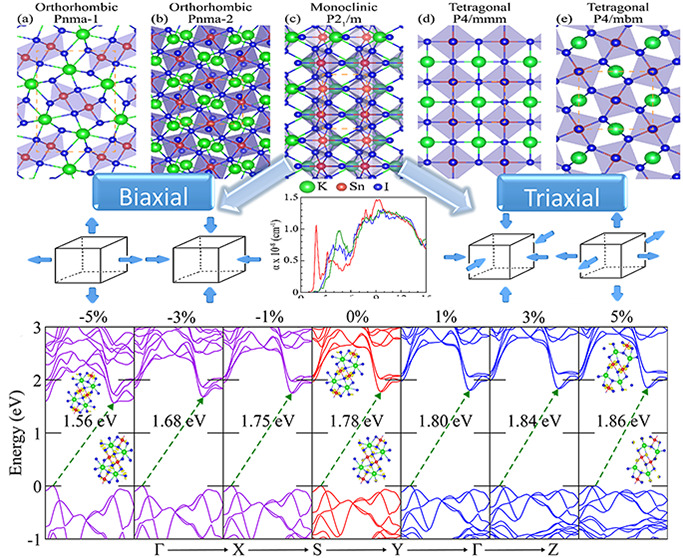

## 1 Introduction

In recent years, rapid progress in photovoltaic and optoelectronic technologies has positioned perovskites as leading candidates due to their direct band gap, strong light absorption, high carrier mobility, low cost, and facile synthesis.^1^ An ideal perovskite, *A* ^+^ *B* ^2+^ *X*_3_^–^ adopts a cubic *Pm3m* structure, analogous to calcium titanate (CaTiO_3_). The crystal lattice consists of corner-sharing BX_6_ octahedra, while the A-site cation occupies the cuboctahedral voids.^2^ The BX_6_ octahedron plays a central role in determining the optical and electronic characteristics of the materials.^3^ Depending on the X-site, perovskites are classified as oxides (ABO_3_) or halides (ABX_3_; X = F, Cl, Br, I), offering broad compositional flexibility.^4^ Their synthesis in diverse morphologies, such as thin films, nanoparticles, nanorods, nanocrystals, and bulk single crystals,^5^ enables applications in solar cells, piezoelectric devices,^5^ optical lenses,^6^ lasers,^7^ high-energy radiation detectors,^8^ and various optoelectronic technologies.^9,10^

Following the discovery of the first organic–inorganic lead-based perovskite CH_3_NH_3_PbI_3_ in 1978 ^11^ and its subsequent use as an absorber layer in solar cells,^12^ research on halide perovskites has grown rapidly. Lead-based hybrid halide perovskites have achieved power conversion efficiencies (PCEs) above 24% at the laboratory scale, making them strong alternatives to silicon devices.^13,14^ However, device stability remains a challenge due to degradation induced by water, oxygen, radiation, and temperature.^14^ Replacing organic cations with monovalent inorganic cations significantly enhances stability, as demonstrated for CsSnBr_3_, which shows nearly an order of magnitude higher stability than that of MASnBr_3_. Beyond stability, the toxicity of lead motivates the substitution of less toxic cations, such as Sn and Ge, leading to the development of all-inorganic, lead-free perovskites as promising candidates for stable and efficient optoelectronic devices.^13–15^

In recent years, numerous theoretical studies have focused on all-inorganic lead-free cubic halide perovskites, exploring their structural stability, electronic, optical, thermoelectric, and mechanical properties.^16–32^ Although CsSnBr_3_ crystallizes in a cubic *Pm3m* structure at room temperature,^33–35^ most halide perovskites retain the cubic phase only at elevated temperatures. CsGeCl_3_ remains cubic (*Pm3m*) at 449 K but transforms into a trigonal phase (*R3m* space group) at 294 K,^36^ while CsGeX_3_ (X = Cl, Br, I) adopts the *R3m* phase at room temperature.^37^ Moreover, many halide perovskites exhibit temperature-dependent phase transitions; for example, RbPbF_3_ transforms from orthorhombic (*Pnma*) at 297 K to cubic (*Pm3m*) at 515 K,^38^ while CsSnBr_3_ and CsSnI_3_ show sequential transitions among orthorhombic, tetragonal, and cubic phases with increasing temperature.^33,39,40^ Theoretical studies further reveal that CsPbI_3_ forms a cubic structure only at high temperatures, specifically around 583 K.^41,42^ More recently, the synthesis of KSnCl_3_ at room temperature revealed the orthorhombic *Pnma* phase,^43^ while the experimental characterization of its iodine analogue, KSnI_3_, remains unreported.

Following the experimental progress on halide perovskites, several theoretical investigations have focused on ABX_3_ structures (A = Li, Na, K, Rb, Cs; B = Pb, Sn, Ge; X = F, Cl, Br, I) in cubic, tetragonal, and orthorhombic phases to examine their electronic features and assess their potential for optoelectronic applications. The reported band gaps of KSnI_3_ vary widely depending on the crystal phase, ranging from 0.40 eV in the cubic and 0.97 eV in the tetragonal structures to between 1.55 and 3.37 eV in the orthorhombic phases.^32^ High-throughput and Goldschmidt tolerance factor analyses have been employed to predict the structural stability of ABX_3_ compounds, identifying potential structures with band gaps of 1.0 and 1.9 eV suitable for tandem solar-cell applications.^16,34^ The orthorhombic (*Pnma-1*) KSnI_3_ exhibits a band gap of 1.84 eV and a simulated solar-cell efficiency approaching 9.8%.^44,45^ In addition, for single-junction perovskite solar cells, the band gap is the most critical parameter influencing the Shockley-Queisser (SQ) efficiency limit, with an optimal range of 1.1–1.4 eV.^46^ Recent advances in perovskites suggest that compositional engineering, dimensional reduction, and strain tuning are effective strategies for optimizing both the band gap and dielectric response.^47^

Band gap engineering plays a crucial role in improving the optoelectronic and photovoltaic performance of perovskite materials. In this study, the structural, vibrational, electronic, optical, and mechanical properties of KSnI_3_ are systematically investigated in its orthorhombic, monoclinic, and tetragonal phases. Additionally, the effects of biaxial and triaxial strains on the electronic and optical properties are examined to identify suitable band gaps for photovoltaic and optoelectronic applications. To the best of our knowledge, such strain effects on KSnI_3_ have not been reported to date. The rest of the article is organized as follows: Section 2 describes the computational methodology. Section 3 presents results and a discussion. Section 3.1 analyzes the structural, vibrational, electronic, and optical properties of the orthorhombic (*Pnma-1* and *Pnma-2*), monoclinic (*P2*_1_/*m*), and tetragonal (*P4/mmm* and *P4/mbm*) phases. Section 3.2 focuses on the electronic and optical properties of stable phases under ±5% biaxial and triaxial strain. Finally, Section 4 summarizes the main conclusions.

## 2 Computational Methodology

In this study, first-principles calculations based on density functional theory (DFT) were performed using the Vienna Ab initio Simulation Package (VASP) to investigate the structural, vibrational, electronic, optical, and mechanical properties of the KSnI_3_ phases.^48,49^ The electron–ion interaction was defined using the projector augmented wave (PAW).^50^ In the geometry optimizations, the generalized gradient approximation (GGA) of the Perdew–Burke–Ernzerhof (PBE) form was considered for the exchange and correlation interaction.^51^ van der Waals (vdW) corrections were included using Becke-Johnson damping of the DFT-3 method.^52^ For the electronic band structure calculations, spin–orbit coupling (SOC) was included within the GGA framework.

The kinetic energy cutoff was set to 500 eV to ensure convergence of the plane-wave basis set. The optimization was continued until the energies converged to 1 × 10^–6^ eV, and the residual pressure on the unit cell was minimized to less than 1 kBar. Gaussian smearing was employed in the total energy calculations, with a smearing width of 0.05 eV. For Brillouin zone integrations, Monkhorst–Pack *k*-point meshes of 8 × 4 × 2, 7 × 8 × 5, 8 × 6 × 6, 6 × 6 × 6, and 6 × 6 × 8 were employed for the orthorhombic (*Pnma-1* and *Pnma-2*), monoclinic (*P2*_1_/*m*), and tetragonal (*P4/mmm* and *P4/mbm*) phases, respectively. The *k*-point grids were selected to ensure a consistent sampling density in reciprocal space across different unit cell sizes, taking into account the lattice parameter ratios of each phase. Convergence tests confirmed that the chosen meshes provided well-converged results.

The Bader technique^53^ was used to analyze the charge transfers in the structures. In order to verify the dynamical stability, phonon band dispersions were calculated using the finite-displacement method, as implemented in the PHON code^54^ and the PHONOPY package.^55,56^ Ab initio quantum molecular dynamics simulations (QMD) were performed to investigate the thermal stability of the structures at a finite temperatures. The simulations were carried out in the canonical (NVT) ensemble for 5 ps at room temperature (300 K) by using a Nosé-Hoover thermostat. A time step of 2 fs was used for numerical integration. Moreover, the small-displacement method was employed to calculate the elastic stiffness tensors of each structure. The optical calculations were performed using the Random Phase Approximation (RPA) method, in conjunction with the PBE functional.^57^

## 3 Results and Discussion

### 3.1 Structural, Electronic, and Dynamic Stability of KSnI_3_ Phases

The optimized atomic structures of the orthorhombic *Pnma-1* and *Pnma-2* (K_4_Sn_4_I_12_), monoclinic *P2*_1_/*m* (K_2_Sn_2_I_6_), tetragonal *P4/mmm* (KSnI_3_), and tetragonal *P4/mbm* (K_2_Sn_2_I_6_) phases are presented in Figure 1a–e. The lattice parameters, cohesive energies, Bader charge transfers, lattice volumes, and interaxial angles of the investigated phases are summarized in Table 1. In all structures, the angle between *z⃗* and *y⃗* is defined as α, the angle between *z⃗* and *x⃗* is defined as β, and the angle between *x⃗* and *y⃗* is defined as γ. Geometry optimization reveals that the orthorhombic phases (20 atoms/unit cell) satisfy *a* ≠ *b* ≠ *c* with α = β = γ = 90°, while the monoclinic *P2*_1_/*m* phase (10 atoms/unit cell) yields *a* ≠ *b* ≠ *c* with α = γ = 90° and β = 111.6°. The tetragonal phases, *P4/mmm* (5 atoms/unit cell) and *P4/mbm* (10 atoms/unit cell), satisfy *a* = *b* ≠ *c* with α = β = γ = 90°. An ideal perovskite structure forms a three-dimensional (3D) framework of corner-sharing BX_6_ octahedra, with the A-site cation occupying cuboctahedral voids in the *Pm3m* space group.^2,7^ The term “distorted perovskite” refers to structures in which the A-site cation is displaced from the center, leading to tilted corner-sharing BX_6_ octahedra. In contrast, “post-perovskite” describes structures that crystallize in one-dimensional (1D) or two-dimensional (2D) edge-sharing octahedral networks. Accordingly, the tetragonal *P4/mmm*, *P4/mbm*, and orthorhombic *Pnma-2* phases are classified as distorted perovskites with corner-sharing BX_6_ octahedra forming a 3D framework. In contrast, the orthorhombic *Pnma-1* phase corresponds to a post-perovskite crystal, consisting of 1D double chains of edge-sharing BX_6_ octahedra. The monoclinic *P2*_1_/*m* phase also exhibits a post-perovskite-type structure, with BX_6_ octahedra corner-sharing along the “*x⃗* ” direction and edge-sharing along the “*y⃗*” direction.

**Figure 1:**
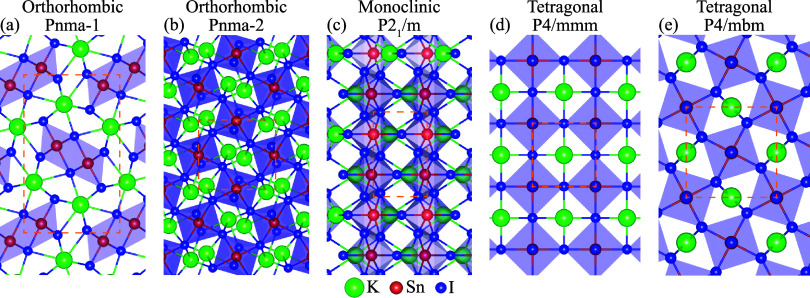
Top views of the optimized crystal structures: (a) K_4_Sn_4_I_12_ in the orthorhombic *Pnma*-1 phase, (b) K_4_Sn_4_I_12_ in the orthorhombic *Pnma*-2 phase, (c) K_2_Sn_2_I_6_ in the monoclinic *P2*_1_/*m* phase, (d) KSnI_3_ in the tetragonal *P4/mmm* phase, and (e) K_2_Sn_2_I_6_ in the tetragonal *P4/mbm* phase. Potassium (K), tin (Sn), and iodine (I) atoms are represented by green, red, and blue colors, respectively.

**Table 1:** For the KSnI_3_ Phases, the Optimized Lattice Parameters (*a*, *b*, and *c*); Angles (α, β, and γ); Lattice Volumes; the Amount of Donated Electrons for K and Sn (ρ_K_ and ρ_Sn_); the Amount of Received Electrons for I (ρ_*I*_); the Cohesive Energies Per Atom (*E*_coh_); and the Electronic Band Gap Energy (*E*_g_)

		lattice parameters	angles	volume	ρ_K_	ρ_Sn_	ρ_I_	cohesive energy	*E*_g_
compounds	phase	(Å)	(deg)	(Å^3^)	(e^–^)	(e^–^)	(e^–^)	(eV/atom)	(eV)
K_4_Sn_4_I_12_	*Pnma-1*	*a* = 4.64	*∝* = 90	797	–0.8	–0.9	0.6	2.78	1.78
		*b* = 10.12	β = 90						
		*c* = 16.97	γ = 90						
K_4_Sn_4_I_12_	*Pnma-2*	*a* = 8.81	*∝* = 90	823	–0.8	–0.9	0.6	2.77	0.82
		*b* = 7.90	β = 90						
		*c* = 11.83	γ = 90						
K_2_Sn_2_I_6_	*P2/mmm*	*a* = 6.21	*∝* = 90	421	–0.8	–0.9	0.6	2.74	1.47
		*b* = 8.70	β = 111.6						
		*c* = 8.39	γ = 90						
KSnI_3_	*P4/mmm*	*a* = 6.59	*∝* = 90	265	–0.8	–0.7	0.5	2.60	0.12
		*b* = 6.59	β = 90						
		*c* = 6.11	γ = 90						
K_4_Sn_4_I_12_	*P4/mbm*	*a* = 8.40	*∝* = 90	439	–0.9	–0.9	0.6	2.73	0.29
		*b* = 8.40	β = 90						
		*c* = 6.22	γ = 90						

The presence of multiple polymorphs in KSnI_3_ indicates significant structural flexibility within the perovskite framework, which is governed by the size and geometric compatibility of the constituent ions. The Goldschmidt tolerance factor (τ) and octahedral factor (μ) are therefore evaluated using effective ionic radii from the literature (*r*_K_ = 164 pm, *r*_Sn_ = 118 pm, and *r*_I_ = 220 pm).^58,59^ The octahedral factor is defined as μ = *r*_B_/*r*_X_, while the tolerance factor is calculated using τ = (*r*_A_ + *r*_X_)/√2­(*r*_B_ + *r*_X_), where *r*_A_, *r*_B_, and *r*_X_ correspond to the ionic radii of the A-site (K), B-site (Sn), and X-site (I) ions, respectively. The calculated octahedral factor, μ = 0.54, falls within the accepted stability range (0.41–0.90), confirming the geometric compatibility of the SnI_6_ octahedra. In addition, the tolerance factor, τ = 0.80, lies at the lower limit of the conventional stability window for halide perovskites, indicating a deviation from the ideal cubic geometry (τ ≈ 1). This reduced tolerance factor suggests that the K cation is relatively small for the iodide framework, which promotes octahedral tilting and structural distortion, and thus intrinsically favors low-symmetry structures.^60^

The differences in octahedral connectivity and structural symmetry among the polymorphs are reflected in their lattice parameters. For the orthorhombic *Pnma-1* phase, the lattice parameters are found to be *a* = 4.64, *b* = 10.12, and *c* = 16.97 Å. The *Pnma-2* phase with the same stoichiometry exhibits expansion along all directions, yielding lattice parameters of *a* = 8.81, *b* = 7.90, and *c* = 11.83 Å. The monoclinic *P2*_1_/*m* phase is characterized by *a* = 6.21, *b* = 8.70, and *c* = 8.39 Å, with β deviating from 90° (111.6°), confirming its lower symmetry. The tetragonal *P4/mmm* phase displays equal lattice parameters along the *x* and *y* directions (*a* = *b* = 6.59 Å) and a shorter lattice constant along the *z* direction (*c* = 6.11 Å), consistent with its tetragonal symmetry. In contrast, the tetragonal *P4/mbm* phase yields *a* = *b* = 8.40 Å and *c* = 6.22 Å, indicating a larger basal plane compared to the *P4/mmm* phase. Overall, the orthorhombic phases exhibit the largest *c* values, while the tetragonal phases are characterized by equal *a* and *b* parameters. The monoclinic structure represents an intermediate case exhibiting moderate lattice dimensions and a characteristic deviation in the β angle. In addition, the cohesive energies (*E*_coh_) of all phases are calculated to evaluate the chemical stability and bonding strength between K, Sn, and I atoms. The cohesive energy, defined as the energy required to separate a crystal into its constituent free atoms, is calculated on a per-atom basis using the following equation:

1 $$E_{\mathrm{coh}}=\frac{1}{x+y+z}\left[\left({\mathrm{xE}}_{K}+{\mathrm{yE}}_{\mathrm{Sn}}+{\mathrm{zE}}_{I}\right)-E_{\mathrm{structure}}\right]$$

Here, *E*_structure_ denotes the total energy of the system, and *E*_K_, *E*_Sn_, and *E*_I_ represent the energies of isolated K, Sn, and I atoms, respectively. The parameters *x*, *y*, and *z* correspond to their respective numbers in the unit cell. As shown in Table 1, *Pnma-1* exhibits the highest cohesive energy of 2.78 eV/atom, followed closely by the *Pnma-2* phase with 2.77 eV/atom, indicating strong bonding interactions among all of the investigated phases. The *P2*_1_/*m* phase has a slightly lower cohesive energy of 2.74 eV/atom. The tetragonal phases yield comparatively smaller values, with 2.73 eV/atom for *P*4/*mbm* and 2.60 eV/atom for *P*4/*mmm*. Overall, the trend in cohesive energy follows the order: *Pnma-1* > *Pnma-2* > *P2*_1_/*m* > *P4/mbm* > *P4/mmm*. The cohesive energy ordering reflects the relative thermodynamic stability of the phases, with *Pnma-1* being the most stable and *P4/mmm* the least stable. However, the very small energy differences among *Pnma-1*, *Pnma-2*, *P2*_1_/*m*, and *P4/mbm* (≈0.01–0.05 eV/atom) indicate a near-degenerate and thermodynamically competitive energy landscape, suggesting that phase coexistence under experimental conditions may occur. Such coexistence is particularly relevant in thin-film or polycrystalline environments where local strain, defects, and growth kinetics can stabilize competing polymorphs. In addition, the small energy separations indicate that phase transitions can be readily triggered by moderate external perturbations such as temperature, strain, or pressure during synthesis and device operation.^61–63^ Moreover, Bader charge analysis reveals that charge transfer predominantly occurs from K and Sn atoms toward I atoms in all of the investigated phases. On average, each K and Sn atom donates about 0.8–0.9 and 0.7–0.9 e^–^, respectively, while the I atoms gain approximately 0.5–0.6 e^–^, reflecting the higher electronegativity of iodine. The nearly uniform charge transfer values across the phases highlight the covalent contribution to the bonding character of K–I and Sn–I interactions, where K and Sn act as electron donors and I atoms act as electron acceptors.

To complement the thermodynamic stability analysis, the dynamic stability of the bulk KSnI_3_ phases is evaluated by calculating their phonon band dispersions along the high-symmetry points of the Brillouin zone (BZ), as illustrated in Figure 2a–e. The phonon spectra indicate that *Pnma-1*, *Pnma-2*, and *P2*_1_/*m* phases are dynamically stable, with no imaginary frequencies across the BZ. In contrast, the tetragonal *P4/mmm* and *P4/mbm* phases display branches with imaginary frequencies, indicating lattice instabilities despite having cohesive energies comparable to those of the stable phases. These instabilities arise from energetically favorable octahedral tilting distortions that are suppressed in the high-symmetry tetragonal framework. The system evolves along symmetry-lowering pathways toward orthorhombic and monoclinic structures, where the tilting patterns are fully stabilized. As a result, the tetragonal phases correspond to saddle points on the potential energy surface rather than true local minima. The phonon spectra exhibit 60 vibrational branches (3 acoustic and 57 optical) for the 20-atom orthorhombic phases, 30 branches (3 + 27) for the 10-atom monoclinic *P2*_1_/*m* and tetragonal *P4/mbm* phases, and 15 branches (3 + 12) for the 5-atom tetragonal *P4/mmm* phase. In addition, the highest optical phonon frequencies at the Γ point are found to be 4.34, 3.81, and 3.80 THz for the dynamically stable *Pnma-1*, *Pnma-2*, and *P2*_1_/*m* phases, respectively. The slightly higher maximum phonon frequency of *Pnma-1* implies stronger interatomic bonding and higher dynamic stiffness. Moreover, the minor phonon softening and increased mode mixing calculated in *Pnma-2* suggest enhanced anharmonicity, which could influence its thermal transport and dielectric behavior. The high-frequency optical modes are mainly associated with Sn–I vibrations, consistent with the characteristic behavior of halide perovskites, where the halogen atoms contribute significantly to the upper optical branches through strong metal–halogen (B–X) bonding.

**Figure 2:**
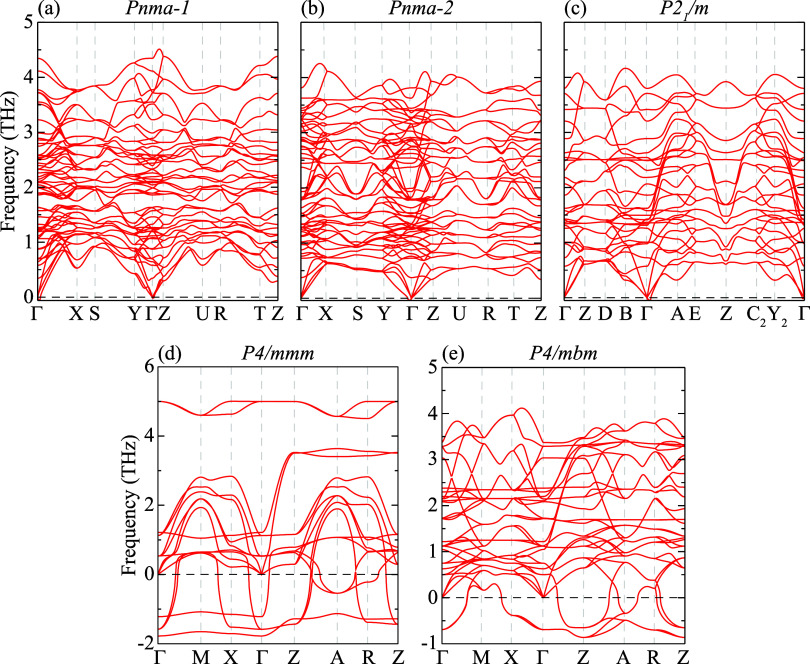
Phonon band dispersions of (a) K_4_Sn_4_I_12_ in the orthorhombic *Pnma*-1 phase, (b) K_4_Sn_4_I_12_ in the orthorhombic *Pnma*-2 phase, (c) K_2_Sn_2_I_6_ in the monoclinic *P2*_1_/*m* phase, (d) KSnI_3_ in the tetragonal *P*4/*mmm* phase, and (e) K_2_Sn_2_I_6_ in the tetragonal *P*4/*mbm* phase.

The finite-temperature stability of the *Pnma-1*, *Pnma-2*, and *P2*_1_/*m* phases of KSnI_3_ is further investigated by performing ab initio QMD simulations at room temperature (300 K). The corresponding time evolution of the total energies is presented in Figure 3. For the 60-atom supercells of the *Pnma-1* and *Pnma-2* phases, the total energy fluctuations are 1.21 and 0.98 meV/atom, respectively, while a fluctuation of 0.96 meV/atom is found for the 80-atom *P2*_1_/*m* supercell. In all cases, the total energy is confined within a narrow oscillation range of approximately ±1 meV/atom, indicating that thermal perturbations around the equilibrium configuration remain minimal throughout the simulation and thus confirm the thermal stability of all three phases. The nearly identical fluctuation amplitudes further support their comparable cohesive energies and close energetic competition.

**Figure 3:**
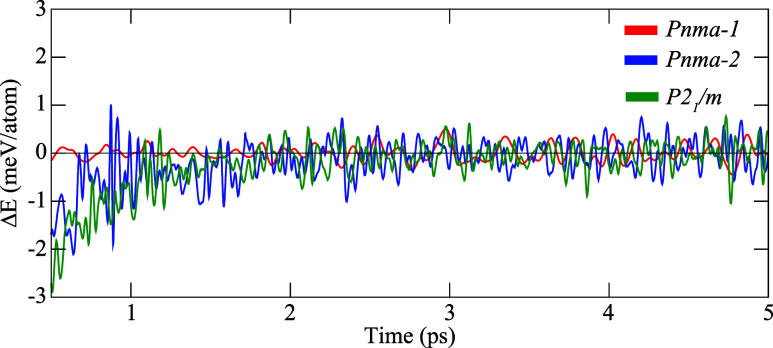
Time evolution of total energy fluctuations at room temperature for the dynamically stable *Pnma-1*, *Pnma-2*, *and P2*_1_/*m* phases of KSnI_3_.

Having established the dynamical and finite-temperature stability of the *Pnma-1*, *Pnma-2*, and *P2*_1_/*m* phases, their mechanical response is quantified through the elastic stiffness tensors (*C_ij_*). The calculated *C_ij_* values satisfy the Born stability criteria for orthorhombic and monoclinic symmetries^64^ (see the Supporting Information), confirming their mechanical stability. Beyond stability, the elastic constants reveal anisotropic behavior with distinct phase-dependent characteristics (Table 2). In particular, the *Pnma-2* phase exhibits the highest longitudinal response along the a- and c- directions (*C*_11_, *C*_33_), indicating enhanced rigidity compared to *Pnma-1*. In contrast, the *P2*_1_/*m* phase is characterized by a dominant stiffness along the *b*-axis while showing relatively weaker resistance along the remaining directions.

**Table 2:** Elastic Stiffness Matrix Elements (*C_ij_*) of KSnI_3_ in *Pnma-1*, *Pnma-2*, *and P2*_1_/*m* Phases

*C_ij_* (GPa)	orthorhombic *Pnma-1*	orthorhombic *Pnma-2*	monoclinic *P2*_1_/*m*
*C*_11_	26.5	33.3	21.3
*C*_12_	13.6	15.0	8.1
*C*_13_	12.8	9.9	7.7
*C*_15_	0	0	–0.1
*C*_22_	17.8	15.4	47.6
*C*_23_	13.8	7.8	5.6
*C*_25_	0	0	2.8
*C*_33_	25.4	29.2	13.1
*C*_35_	0	0	–0.2
*C*_44_	12.5	11.1	5.2
*C*_46_	0	0	0.1
*C*_55_	5.8	3.7	2.3
*C*_66_	8.6	7.0	3.0

Building upon such anisotropic elastic behavior, the mechanical response is further characterized through the derived elastic moduli, including the Young’s modulus (*E*), shear modulus (*G*), and Poisson’s ratio (*v*). As summarized in Table 3, the directional Young’s modulus reveals a pronounced anisotropic stiffness distribution across all phases. For the *Pnma-1* phase, moderate stiffness is calculated along the *x*- and *z*-directions (15.8 and 14.4 GPa), while the *y*-direction remains comparatively soft (8.2 GPa). The *Pnma-2* phase exhibits enhanced rigidity, most prominently along the *z*-axis (24.9 GPa), indicating a stronger resistance to uniaxial deformation along the *z*-axis. In contrast, the *P2*_1_/*m* phase displays a markedly elevated Young’s modulus along the *y*-axis (40.3 GPa), significantly exceeding that of the orthorhombic phases, while the *x*- and *z*-directions remain considerably softer (16.2 and 10.1 GPa).

**Table 3:** Derived Elastic Moduli of KSnI_3_ in the *Pnma-1*, *Pnma-2*, *and P2*_1_/*m* Phases: Young’s Moduli (*E*), Shear Moduli (*G*), and Poisson Ratios (*v*)

elastic moduli	orthorhombic *Pnma-1*	orthorhombic *Pnma-2*	monoclinic *P2*_1_/*m*
*E*_1_ (GPa)	15.8	18.4	16.2
*E*_2_ (GPa)	8.2	8.1	40.3
*E*_3_ (GPa)	14.4	24.9	10.1
*G*_23_ (GPa)	12.5	11.1	5.1
*G*_31_ (GPa)	5.8	3.7	2.1
*G*_12_ (GPa)	8.6	7.0	3.0
*v*_21_	0.32	0.41	0.28
*v*_31_	0.14	0.12	0.33
*v*_23_	0.38	0.13	0.29

The shear moduli quantify the resistance to angular distortions across the different crystallographic planes. The orthorhombic phases, particularly *Pnma-1*, exhibit relatively high shear rigidity, as reflected by the large G_23_ value (12.5 GPa), indicating strong resistance against shear distortions in the yz-plane. The *Pnma-2* phase follows a similar trend with slightly reduced shear components. In contrast, the *P2*_1_/*m* phase displays relatively low shear rigidity with all shear components falling below 6 GPa, showing a significantly reduced resistance to angular deformation. Overall, the orthorhombic phases exhibit a more balanced yet anisotropic elastic response, whereas the monoclinic phase combines pronounced directional stiffness with weakened shear resistance, leading to a distinctly enhanced anisotropic mechanical response.

Poisson ratio (*ν*) describes the transverse strain response under uniaxial loading, defined as the negative ratio of lateral to axial strain. Larger values indicate stronger elastic coupling between the orthogonal directions. As summarized in Table 3, the *Pnma-1* phase shows pronounced coupling involving the *y*-axis (*ν*_21_ = 0.32, *ν*_23_ = 0.38), while the relatively small *ν*_31_ value of 0.14 reflects weak coupling between the *z*- and *x*-directions. In contrast, the *Pnma-2* phase exhibits enhanced coupling primarily within the *xy* plane, where *ν*_21_ reaches 0.41, whereas *ν*_31_ and *ν*_23_ remain low (0.12 and 0.13), indicating strongly anisotropic and plane-dependent deformation behavior. The monoclinic *P2*_1_/*m* phase displays a more balanced distribution of Poisson ratios (*ν*_21_ = 0.28, *ν*_23_ = 0.29, and *ν*_31_ = 0.33), suggesting a more evenly distributed transverse strain response than in the orthorhombic phases, although the overall elastic behavior remains anisotropic.

In order to analyze the electronic behavior in momentum space, the band structures and corresponding partial density of states (PDOS) of the *Pnma-1*, *Pnma-2*, *P2*_1_/*m*, *P4/mmm*, and *P4/mbm* phases of KSnI_3_ are calculated across the entire BZ, as shown in Figure 4a–e. The high-symmetry *k*-point paths are selected according to the crystal symmetry of each phase, namely Γ-X-S-Y-Γ-Z-U-R-T-Z for the orthorhombic structures (*Pnma-1* and *Pnma-2*), Γ-Z-D-B-Γ-A-E-Z-C_2_-Y_2_-Γ for the monoclinic phase (*P2*_1_/*m*), and Γ-M-X-Γ-Z-A-R-Z for the tetragonal phases (*P4/mmm* and *P4/mbm*). All calculations include SOC, which plays a crucial role due to the presence of heavy elements. The calculated band structures reveal both direct and indirect semiconducting characteristics among the five phases. The *P4/mmm* and *P4/mbm* phases exhibit direct band gaps of 0.12 and 0.29 eV at the A and Z points, respectively. The *Pnma-2* phase also displays a direct band gap of 0.82 eV at the Γ point, suggesting the potential suitability for optoelectronic applications, particularly in the infrared regime. In contrast, the *Pnma-1* and *P2*_1_/*m* phases are indirect semiconductors, with valence-band maximum (VBM) located between Γ-X and C_2_–Y_2_, and conduction-band minimum (CBM) at Y and Y_2_, respectively. The corresponding band gap values of 1.78 eV for *Pnma-1* and 1.47 eV for *P2*_1_/*m* fall within an energy range suitable for optoelectronic and photovoltaic applications. The difference between the direct band gap in *Pnma-2* and the indirect band gap in *Pnma-1* originates from their distinct octahedral connectivity. The three-dimensional corner-sharing network in *Pnma-2* enhances orbital hybridization and band dispersion, allowing the VBM and CBM to align at the same *k*-point, whereas the edge-sharing chains in *Pnma-1* lead to reduced orbital overlap, more localized electronic states, and less dispersive bands, resulting in an indirect band gap character. Although the tetragonal phases exhibit significantly narrower band gaps, well below the range typically considered suitable for efficient solar energy conversion, they may still be promising for alternative optoelectronic applications such as infrared detection or as subcells in tandem solar architectures. In addition, the progressive narrowing of the band gap from orthorhombic to tetragonal phases reflects reduced octahedral distortion and enhanced orbital overlap between neighboring Sn–I units, consistent with symmetry-driven electronic delocalization. Importantly, the tetragonal phases are dynamically unstable at 0 K. However, in halide perovskites, high-symmetry phases are frequently realized as metastable or strain-stabilized states under finite temperature, pressure, or substrate-induced constraints.^61–63^ Therefore, despite their intrinsic instability in bulk equilibrium, tetragonal phases remain physically meaningful and potentially realizable configurations with favorable electronic characteristics, particularly in strain-engineered or thin-film optoelectronic platforms.

**Figure 4:**
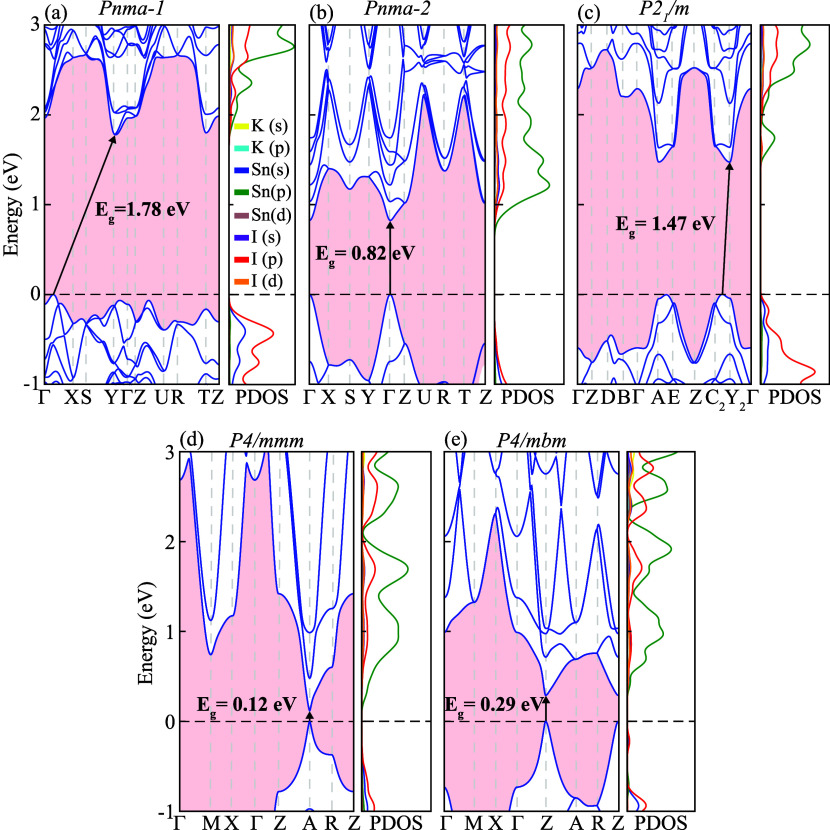
Electronic band structures and corresponding partial density of states of (a) K_4_Sn_4_I_12_ in the orthorhombic *Pnma*-1 phase, (b) K_4_Sn_4_I_12_ in the orthorhombic *Pnma*-2 phase, (c) K_2_Sn_2_I_6_ in the monoclinic *P2*_1_/*m* phase, (d) KSnI_3_ in the tetragonal *P*4/*mmm* phase, and (e) K_2_Sn_2_I_6_ in the tetragonal *P*4/*mbm* phase.

The PDOS analysis further clarifies the electronic structure by revealing the orbital contributions near the band edges. In all phases, the VBM is primarily composed of I-*p* and Sn-*s* orbitals, while the CBM mainly originates from the Sn-*p* states with a secondary contribution from I-*p* orbitals. Potassium orbitals contribute negligibly near the band edges, consistent with its role as an ionic donor. Such an orbital distribution highlights the dominant influence of the SnI_6_ octahedral framework, where strong *p*-*s* and *p*-*p* hybridizations between Sn and I atoms govern the electronic transitions. The alignment of these states near the Fermi level explains the strong absorption in the visible and ultraviolet regions reported for the stable phases, thereby supporting the potential of KSnI_3_ perovskites in optoelectronic and photovoltaic applications.

Following the electronic analysis, the optical responses of dynamically stable *Pnma-1*, *Pnma-2*, and *P2*_1_/*m* phases of KSnI_3_ are systematically analyzed in the photon energy range of 0–15 eV to understand their light-matter interactions. Due to the inherent anisotropy of the crystal structures, the optical features are resolved along different crystallographic directions (*x*, *y*, and *z*) to capture the directional dependence of light-matter interactions. The absorption coefficients (α­(ω)), refractive indices (*n*(ω)), and reflectance spectra (*R*(ω)) are obtained from the frequency-dependent complex dielectric function (ε­(ω)), which can be expressed as

2 $$\varepsilon \left(\omega \right)=\varepsilon_{1}\left(\omega \right)+i\varepsilon_{2}\left(\omega \right)$$

Here, ε_1_(ω) denotes the real part related to polarization under photon excitation, while ε_2_(ω) represents the imaginary part associated with interband absorption and energy dissipation. The static dielectric constant (ε_1_(0)) corresponds to the zero-energy limit and reflects the intrinsic polarizability of the material. The real and imaginary components of ε­(ω) are further used to calculate the refractive index and extinction coefficient (κ­(ω)) as

3 $$n\left(\omega \right)=1/\sqrt{2}{\left[\varepsilon_{1}+{\left(\varepsilon_{1}^{2}+\varepsilon_{2}^{2}\right)}^{1/2}\right]}^{1/2}$$

4 $$\kappa \left(\omega \right)=\frac{1}{\sqrt{2}}{\left[-\varepsilon_{1}+\sqrt{{\left(\varepsilon_{1}\left(\omega \right)\right)}^{2}+{\left(\varepsilon_{2}\left(\omega \right)\right)}^{2}}\right]}^{1/2}$$

Subsequently, the reflectance and absorption spectra are determined using

5 $$R\left(\omega \right)=\frac{{\left(n-1\right)}^{2}+\kappa^{2}}{{\left(n+1\right)}^{2}+\kappa^{2}}$$

6 $$\alpha \left(\omega \right)=\sqrt{2\omega }{\left[{\left({\left(\varepsilon_{1}\left(\omega \right)\right)}^{2}+{\left(\varepsilon_{2}\left(\omega \right)\right)}^{2}\right)}^{1/2}-\varepsilon_{1}\left(\omega \right)\right]}^{1/2}$$

These interrelated optical constants collectively capture the essential aspects of light-matter interactions across the investigated photon energy range and serve as key indicators for assessing the optoelectronic potential of KSnI_3_-based perovskites. Figures 5 and 6 present the calculated ε_1_(ω) and ε_2_(ω), α­(ω), *n*(ω), and *R*(ω) spectra for the *Pnma-1*, *Pnma-2*, and *P2*_1_/*m* phases. As summarized in Table 4, the values obtained for different photon polarization directions clearly demonstrate the anisotropic optical response of all three structures.

**Figure 5:**
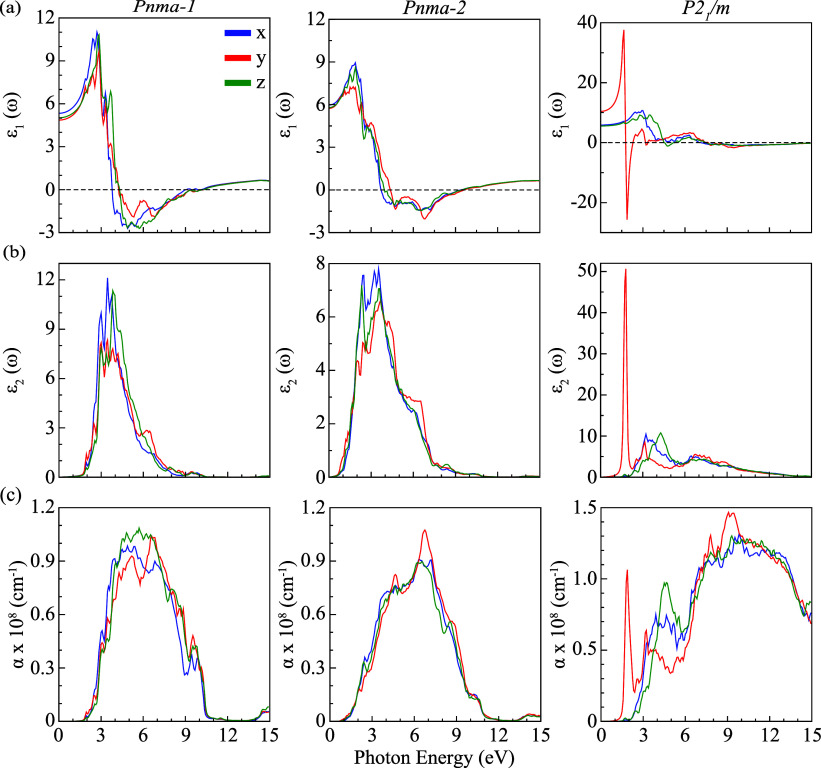
Calculated optical properties: (a) real part of dielectric function ε_1_(ω), (b) imaginary part of dielectric function ε_2_(ω), and (c) absorption coefficient α­(ω) for K_4_Sn_4_I_12_ in the orthorhombic *Pnma-1 and Pnma-2* phases, as well as K_2_Sn_2_I_6_ in the monoclinic *P2*_1_/*m* phase.

**Figure 6:**
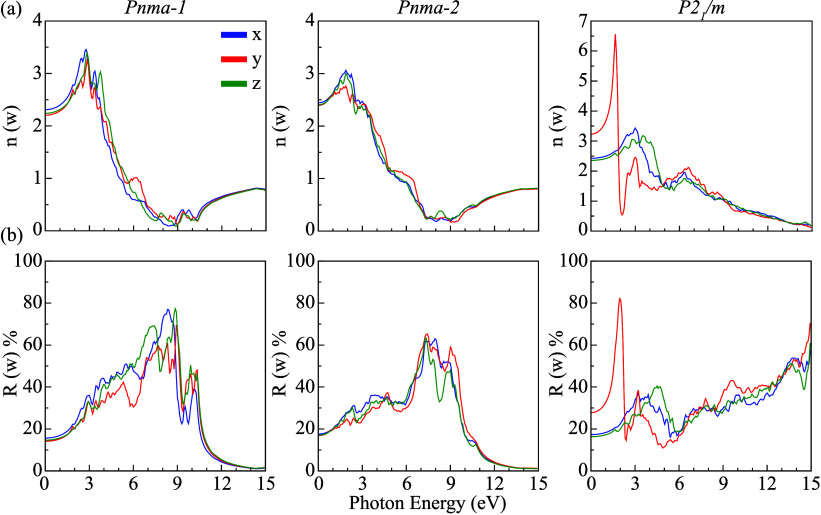
(a) Refractive index *n*(ω) and (b) reflectance *R*(ω) spectra of *Pnma*-1, *Pnma*-2, and *P2*_1_/*m* phases.

**Table 4:** Static Dielectric Constant ε(0), Real Dielectric Constant ε_1_(ω), Imaginary Dielectric Constant ε_2_(ω), Absorption Coefficient α­(ω), Refractive Index *n*(ω), and Reflectivity *R*(ω) Values of the *Pnma-1*, *Pnma-2*, and *P2*_1_/*m* Materials under the Influence of −5%, 0%, and 5% Biaxial Strain

		*Pnma-1*			*Pnma-2*			*P2*_1_/*m*		
		polarization direction			polarization direction			polarization direction		
strain ratio	optic parameters	*x*	*y*	*z*	*x*	*y*	*z*	*x*	*y*	*z*
–5%	ε(0)	6.28	5.88	6.20	6.59	6.59	6.12	6.68	9.31	6.58
	ε_1_(ω)_max_	11.83	10.31	12.30	9.33	8.01	8.87	13.35	23.42	10.43
	ε_2_(ω)_max_	11.96	9.51	13.09	9.18	9.88	7.86	9.02	8.54	13.64
	α(ω)_max_	1.14 × 10^8^	1.21 × 10^8^	1.31 × 10^8^	1.18 × 10^8^	1.25 × 10^8^	1.07 × 10^8^	1.40 × 10^8^	1.59 × 10^8^	1.42 × 10^8^
	*n*(ω)_max_	3.50	3.36	3.65	3.13	2.88	3.03	3.72	4.91	3.44
	*R*(ω)%	80	78	79	72	77	63	66	68	66
0%	ε(0)	5.34	4.86	5.02	5.97	5.73	5.79	5.88	10.43	5.54
	ε_1_(ω)_max_	11.01	9.68	10.82	8.94	7.20	8.59	10.69	37.61	9.23
	ε_2_(ω)_max_	12.12	8.42	11.42	7.80	6.62	7.06	10.46	50.65	10.78
	α(ω)_max_	0.99 × 10^8^	1.04 × 10^8^	1.07 × 10^8^	0.91 × 10^8^	1.07 × 10^8^	0.90 × 10^8^	1.32 × 10^8^	1.47 × 10^8^	1.30 × 10^8^
	*n*(ω)_max_	3.46	3.24	3.39	3.06	2.76	3.00	3.43	6.56	3.18
	*R*(ω)%	77	70	77	63	65	63	58	82	61
5%	ε(0)	4.74	4.09	4.24	5.68	5.29	5.50	5.24	6.41	4.85
	ε_1_(ω)_max_	9.96	7.82	9.17	8.81	6.78	8.48	10.91	22.06	7.92
	ε_2_(ω)_max_	11.06	7.75	11.02	8.07	5.74	6.87	10.06	27.44	8.81
	α(ω)_max_	0.93 × 10^8^	0.87 × 10^8^	1.01 × 10^8^	0.76 × 10^8^	0.79 × 10^8^	0.76 × 10^8^	1.19 × 10^8^	1.31 × 10^8^	1.24 × 10^8^
	*n*(ω)_max_	3.33	2.90	3.09	3.07	2.76	2.95	3.38	4.83	2.97
	*R*(ω)%	74	59	70	64	54	60	51	64	49

The static dielectric constants are calculated as 5.34, 4.86, and 5.02 along the *x*-, *y*-, and *z*-directions for the *Pnma-1* phase; 5.97, 5.73, and 5.79 for the *Pnma-2* phase; and 5.65, 10.43, and 5.54 for the *P2*_1_/*m* phase, respectively. The unusually large ε_1_(0) value along the *y*-axis for *P2*_1_/*m* indicates strong polarizability and a high static refractive index, marking a highly responsive optical axis. The real part of the dielectric function exhibits resonant features in the visible range, with maxima at 2.70/2.85/2.85 eV for *Pnma-1*, 1.88/1.73/1.88 eV for *Pnma-2*, and 3.00/1.65/3.45 eV for *P2*_1_/*m* (see Figure 5a). The exceptionally low-energy y-polarized resonance at 1.65 eV in *P2*_1_/*m*, combined with its large static dielectric constant, highlights its strong anisotropic optical behavior. As shown in Figure 5b, the imaginary part, *ε*_2_(ω), reveals interband transitions with first peak positions corresponding to excitations from I-*p* dominated valence states to Sn/I-*p* hybridized conduction states, consistent with the band-edge orbital analysis.

Building upon the dielectric analysis, we examined the absorption behavior of the structures to clarify their optical activity. The optical band gaps, determined from the first rise in α­(ω), are 1.95, 1.20, and 1.80 eV for *Pnma-1*, *Pnma-2*, and *P2*_1_/*m* (Figure 5c), respectively, in close agreement with our predicted electronic band gaps. Beyond the onset region, all phases display strong absorption across the visible and ultraviolet ranges with intensity modulated by light polarization.

The refractive index *n*(ω) characterizes the transparency and polarizability of a material, with higher values reflecting stronger light-matter interactions and reduced optical transmission. As shown in Figure 6a, the static refractive indices at zero photon energy attain their maximum values along the *x*-direction for the *Pnma*-1 and *Pnma*-2 phases (2.35 and 2.45, respectively), while for the *P2*_1_/*m* phase, the maximum value (3.15) occurs along the *y*-direction. The markedly larger value for *P2*_1_/*m* indicates enhanced polarizability and an optical response, suggesting reduced transparency relative to the orthorhombic phases. Across all structures, maxima in *n*(ω) occur within the visible range, consistent with their electronic band gaps. For comparison, halide perovskites typically exhibit refractive indices in the range of *n* ≈ 2–3 in the visible and near-infrared regions.^65,66^ In contrast, *P2*_1_/*m* exhibits an exceptionally large refractive index of 6.56 under y-polarized excitation in the visible region, highlighting its strong optical confinement capability and potential for photonic applications, where a high refractive index contrast is advantageous. This large value corresponds to a sharp, polarization-dependent peak within a narrow spectral range rather than representing a broadband refractive index, reflecting the strong optical anisotropy of the *P2*_1_/*m* phase.

The reflectivity spectra *R*(ω) of the KSnI_3_ phases are shown in Figure 6b, providing further details on their surface optical response. Reflection peaks at different photon energies originate from anisotropic dielectric screening and interband transition resonances. All three phases exhibit pronounced reflectance in the ultraviolet region, exceeding 70% in certain polarization directions, which could make them promising as reflective or UV-protective coating materials. In addition, *P2*_1_/*m* shows particularly high reflectivity in the visible region, reaching 82% at 1.95 eV under y-polarization. Such strong direction-dependent reflectance highlights the suitability of *P2*_1_/*m* for applications requiring selective optical shielding or wavelength-specific reflection. In contrast, *Pnma-1* and *Pnma-2* display more moderate and isotropic reflectance profiles, which may be advantageous for broadband absorption. Overall, the optical features of KSnI_3_ demonstrate its potential for polarization-sensitive and wavelength-tunable optoelectronic and photonic devices. In particular, the pronounced optical anisotropy of the *P2*_1_/*m* phase enables polarization- and direction-dependent device operation, supporting functionalities such as polarization filtering, anisotropic waveguiding, and polarization-selective optical components along with light-harvesting applications.

### 3.2 Effect of Biaxial and Triaxial Strain on the Structural, Electronic, and Optical Properties of *Pnma*-1, *Pnma*-2, and *P2*_1_/*m* Phases

The structural, electronic, and optical properties of halide perovskites are highly sensitive to external perturbations. In realistic device environments, mechanical strain naturally arises from substrate-induced lattice mismatch, thermal expansion, and thin-film growth processes, making it a key factor in governing the optoelectronic behavior of KSnI_3_. Applying biaxial or triaxial strain modifies the local bonding environment and lattice symmetry, leading to significant changes in electronic band dispersion and optical response. Therefore, for KSnI_3_ in the *Pnma-1*, *Pnma-2*, and *P2*_1_/*m* phases, strain engineering offers an effective route to modulate the band gap and light-matter interaction. In this section, we investigate the effects of compressive and tensile strains ranging from −5% to +5%, a commonly used range in first-principles studies that remains within experimentally accessible limits. In the biaxial strain calculations, the *z* lattice vector is kept fixed, while the *x* and *y* lattice vectors are varied in ± 1% increments. In contrast, under triaxial strain, all three lattice vectors are uniformly strained within the same range.

Within the applied strain range (±5%), all structures converge smoothly during geometry optimization, with no indication of a structural phase transition, symmetry breaking, or abrupt reconstruction. The variations in the lattice parameters under biaxial and triaxial strain are summarized in Tables S1–S6 in the Supporting Information. The structural response of KSnI_3_ phases exhibits systematic trends under both compressive and tensile strain. Across all phases, biaxial strain primarily affects the *a* and *b* lattice parameters, while the *z* direction remains constrained. For the *Pnma-1* phase, the lattice constants *a* and *b* decrease under compression (from 4.64 and 10.12 Å to 4.41 and 9.62 Å at −5%) and increase under tension (up to 4.87 and 10.63 Å at +5%), with *c* fixed at 16.97 Å. The *Pnma-2* phase follows the same behavior, with *a* and *b* varying from 8.81 and 7.90 Å to 8.37 and 7.51 Å under compression and expanding to 9.25 and 8.30 Å under tension, while c remains constant at 11.83 Å. In the *P2*_1_/*m* phase, the lattice parameters *a* and *b* change from 6.21 and 8.70 Å to 5.90 and 8.26 Å under compression and increase to 6.52 and 9.13 Å under tension, with *c* remaining constant at 8.39 Å. In contrast, under a triaxial strain, all lattice parameters evolve simultaneously. For *Pnma-1*, the *c* parameter decreases to 16.12 Å under compression and increases to 17.82 Å under tension. For *Pnma-2*, c varies from 11.24 to 12.42 Å, while for *P2*_1_/*m*, it decreases to 7.97 Å under compression and increases to 8.81 Å under tension. This contrast highlights the fundamental difference between the two strain modes, where biaxial strain enforces anisotropic lattice deformation, whereas triaxial strain enables a more uniform, isotropic structural response.

The application of strain modifies the relative positions of electronic bands, inducing shifts, convergence, or separation of the CBM and VBM. In the *Pnma-1* phase, which retains an indirect band gap character under all strain conditions, the band structures in Figure 7a,b demonstrate distinct strain-dependent responses: biaxial strain causes a nonmonotonic band gap variation, while triaxial strain yields a systematic evolution.

**Figure 7:**
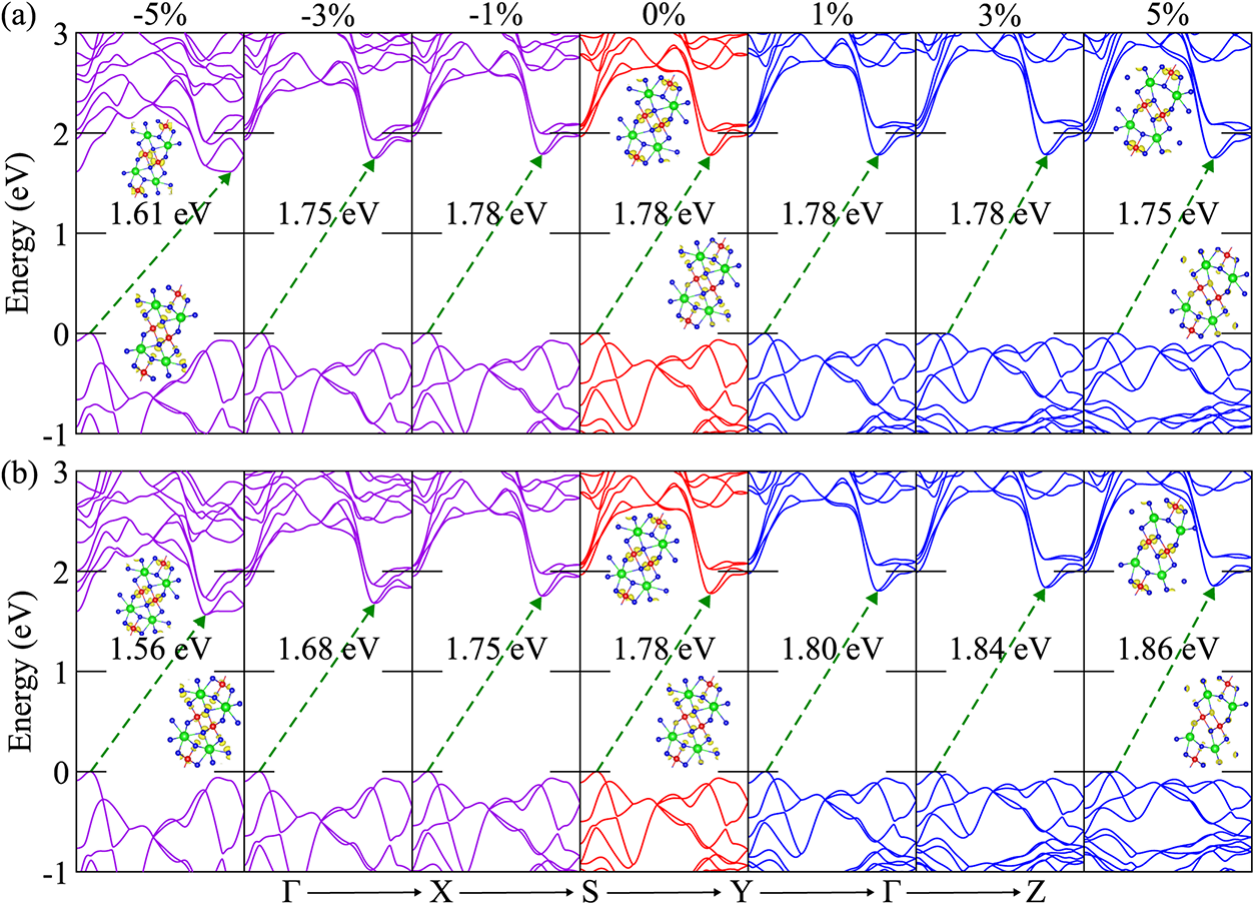
Calculated electronic band structures of the *Pnma-1* phase under (a) biaxial and (b) triaxial strains ranging from −5% to +5%, along with the corresponding atomic orbital contributions to the VBM and CBM states. The isosurface value for the atomic orbitals is taken as 2.3 × 10^–6^ e/Å^3^. K, Sn, and I atoms are represented by green, red, and blue colors, respectively.

Under biaxial strain, the band gap remains nearly unchanged, exhibiting only minor variations. In the compressive regime, it remains at 1.78 eV for 0% and −2% and then decreases to 1.75 eV at −3% and −4%, followed by a more significant reduction to 1.61 eV at −5%. Under tensile strain, the band gap remains nearly constant at 1.78 eV up to +3%, and then slightly decreases to 1.76 and 1.75 eV at +4% and +5%, respectively, indicating a weaker sensitivity to tensile deformation. These trends originate from strain-induced modifications in the orbital hybridization and bonding geometry. At low strain levels, the weak variation reflects only minor perturbations in σ-type Sn-*p*/I-*p* hybridization, resulting in negligible changes in the bonding–antibonding splitting. Consistently, under compression, the nearly unchanged band gap up to −2% is due to the limited variation of both the Sn–I–Sn and I–Sn–I bond angles (86–88° and 176.3–177.7°, respectively), indicating largely preserved orbital overlap. At higher compressive strain, both angles decrease significantly, reaching 80.8° and 170.7° at −5%, reflecting enhanced octahedral tilting. This distortion modifies the Sn–I orbital hybridization, particularly affecting the antibonding CBM states, and leads to band gap narrowing. Under tensile strain, the band gap initially shows little change due to the largely preserved bonding geometry, as the I–Sn–I angle slightly increases from 177.7° toward 179°. At higher strain, the angle decreases back toward 177° and, together with the slight broadening of the bond-length distribution (from 3.03 to 3.30 Å at 0% to 3.03 to 3.40 Å at +5%), modifies the band-edge hybridization, resulting in a slight reduction in the band gap.

Under triaxial strain, the band gap exhibits a monotonic evolution, decreasing under compression and increasing under tensile deformation. Specifically, the gap reduces from 1.78 eV at equilibrium to 1.56 eV at −5%, while it increases steadily to 1.86 eV at +5%, demonstrating systematic tunability of the *Pnma-1* phase. Compared with the biaxial case, the triaxial response is more symmetric and pronounced, reflecting the isotropic perturbation of the three-dimensional bonding network. This behavior is governed by strain-induced modifications in bonding–antibonding interactions: tensile strain enhances the separation, whereas compressive strain reduces it. Consistently, under compression, both the Sn–I–Sn and I–Sn–I bond angles decrease (88° → 82° and 177° → 171°), accompanied by bond shortening (3.03–3.30 Å → 2.95–3.19 Å), which lowers the antibonding CBM and narrows the band gap. In contrast, tensile strain increases the Sn–I–Sn angle (88° → 92°) and elongates the bonds (3.03 Å, 3.30 → 3.08 Å, 3.40 Å), reducing orbital overlap and leading to band gap widening. The ability to tune the band gap highlights the potential of the *Pnma-1* phase for strain-engineered optoelectronic applications, particularly in perovskite-based photovoltaics and light-emitting devices.

Orbital-resolved charge density analysis reveals that the VBM is predominantly composed of localized I-*p* states with minor I-*s* contributions, while the CBM mainly originates from Sn-*p* orbitals with σ-type interactions with I-*p* states along the I–Sn–I direction. Under −5% biaxial strain, the VBM is primarily localized on I atoms with dominant I-*p* character, while the CBM consists of antibonding Sn-*p*/I-*p* states with partial spatial localization. Under +5% biaxial strain, the VBM shows mixed I-*p* and I-*s* contributions, whereas the CBM becomes more confined around Sn atoms and is dominated by Sn-*p* antibonding states with reduced Sn-*p*/I-*p* hybridization. Under −5% triaxial strain, the VBM remains strongly localized on I atoms, while the CBM is formed by σ-type antibonding states arising from the Sn-*p* and I-*p* orbitals. At + 5% triaxial strain, the VBM retains its I-*p* character with emerging I-*s* contributions, whereas the CBM is governed by highly localized Sn-*p* antibonding states, reflecting weakened Sn-*p*/I-*p* hybridization.

For the *Pnma-2* phase, which maintains a direct band gap character under all strain conditions, the gap response exhibits distinct trends under biaxial and triaxial loading. Under biaxial compression, the band gap remains nearly constant at 0.82 eV up to −3% strain, followed by a gradual reduction to 0.78 eV at −5%. Similarly, under biaxial tensile strain, only minor variations are found, with the gap slightly decreasing to 0.81 eV at +5%, indicating weak sensitivity to biaxial tensile deformation (Figure S1a). This behavior originates from anisotropic structural distortions. Under compression, the first Sn–I–Sn angle slightly increases (140° → 141°), while the second decreases markedly (139° → 132°), enhancing octahedral tilting, which is consistent with the band gap reduction. In contrast, under tension, the first angle slightly decreases (140° → 139°), whereas the second increases (139° → 145°), indicating anisotropic and partly compensating structural distortions. As a result, the electronic structure is only weakly affected and the band gap exhibits only a slight reduction. Under triaxial strain, the band gap exhibits a more systematic evolution. Under compressive triaxial strain, the gap decreases gradually from 0.82 to 0.79 eV at −5%, whereas tensile triaxial strain leads to a monotonic increase from 0.82 to 0.85 eV at +5% (Figure S1b). This contrasting behavior arises from the isotropic modification of the 3D Sn–I network. Under compression, both Sn–I–Sn angles decrease significantly (139° → 132° and 140° → 132°, respectively), indicating enhanced octahedral tilting throughout the lattice. This structural distortion shortens the Sn–I bonds and strengthens orbital overlap, lowering the antibonding Sn-*p* CBM and leading to a reduction in the band gap. By contrast, under tensile strain, both angles increase (139° → 146° and 140° → 147°), reflecting a more linear bonding geometry. This reduces orbital overlap and weakens Sn–I hybridization, shifting the antibonding Sn-*p* CBM upward, while the VBM remains comparatively less sensitive to strain, resulting in a monotonic increase in the band gap. Overall, compared to *Pnma-1*, the weaker response of *Pnma-2 to biaxial strain* reflects the more limited sensitivity of its band-edge states to such perturbations.

In the monoclinic *P2*_1_/*m* phase, the strain response differs markedly from that in the orthorhombic phases (Figure S2). Within the ± 5% strain window, the indirect band gap exhibits an overall increasing trend under both biaxial and triaxial loading, with no indication of gap narrowing. Under biaxial strain, the gap increases from 1.47 eV at equilibrium to 1.66 eV at −5% and to 1.80 eV at +5%. A similar trend holds for triaxial strain, where the gap reaches 1.64 eV at −5% and 1.81 eV at +5%. This behavior indicates that the *P2*_1_/*m* electronic structure is resistant to strain-induced band gap reduction within the considered range. Under compression, the Sn–I–Sn angle decreases (biaxial: 167° → 159°; triaxial: 166° → 155°) together with bond shortening, inducing a pronounced octahedral distortion that modifies band-edge coupling and results in a moderate band gap increase. In contrast, under tensile strain, the angular response is modest under biaxial loading (167° → 168°) but more pronounced under triaxial loading (166° → 174°), while substantial elongation of representative Sn–I bonds (biaxial: 3.14 → 3.59 Å; triaxial: up to 3.57 Å for Sn–I) weakens hybridization, leading to a more pronounced band gap increase. The resulting strain-tunable band gap range of 1.47 to 1.81 eV highlights the potential of the *P2*_1_/*m* phase for strain-engineered optoelectronic and photovoltaic applications.

The strain-dependent evolution of the band gap and external pressure for the KSnI_3_ phases is summarized in Figure 8a,b. A systematic decrease in external pressure is calculated across the full deformation range, from compressive (−5%) to tensile (+5%) regimes, reflecting the elastic consistency of the calculations and the strong coupling between the strain and stress response. At −5% biaxial strain, the *Pnma-1* and *P2*_1_/*m* phases yield comparable band gap values of 1.61 and 1.66 eV under external pressures of 2.13 and 2.13 GPa, respectively. Under −5% triaxial strain, the band gaps slightly decrease to 1.56 eV for *Pnma-1* and 1.64 eV for *P2*_1_/*m*, but they are achieved at notably higher pressures of 3.87 and 3.03 GPa. A similar pressure advantage is also found for the *Pnma-2* phase, although the associated band gap variation is much weaker than that in *Pnma-1* and *P2*_1_/*m*. From an application perspective, access to comparable band gap values under reduced pressure conditions improves experimental feasibility and may facilitate device integration. Importantly, under practical device environments where strain is inherently present, mechanical deformation can serve as a controllable tuning parameter, enabling systematic modulation of the electronic properties of KSnI_3_ without chemical modification.

**Figure 8:**
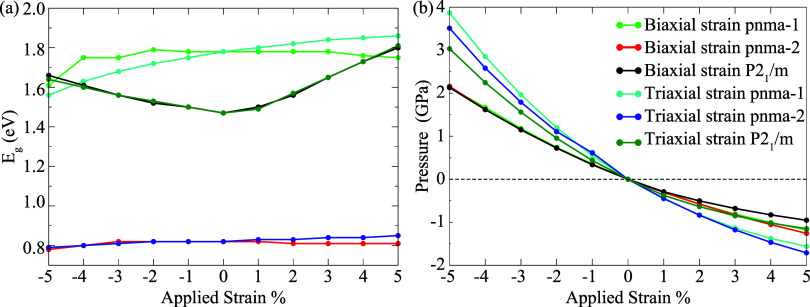
Evolution of (a) electronic band gaps and (b) the corresponding pressures under applied biaxial and triaxial strains (±5%) for the *Pnma-1*, *Pnma-2*, *and P2*_1_/*m* phases.

Overall, the KSnI_3_ phases exhibit distinct strain-dependent electronic responses under biaxial and triaxial loading. For the orthorhombic phases, biaxial strain leads to weak and in some cases nonmonotonic band gap variations, whereas triaxial strain induces a more systematic evolution. By contrast, the monoclinic *P2*_1_/*m* phase exhibits a robust band gap increase under both strain conditions, highlighting its unique electronic response compared to that of the orthorhombic phases. The differences arise from the phase-dependent structural response to strain, where anisotropic lattice distortions modify orbital overlap in different ways, depending on both crystal symmetry and the dimensionality of the applied deformation. These results demonstrate that strain dimensionality plays a critical role in governing the electronic response, providing a controllable pathway for band-structure engineering and the optimization of optoelectronic functionality in KSnI_3_-based systems.

To establish a direct link between strain-induced electronic modifications and optical response, the evolution of the refractive index, the dielectric function, absorption coefficient, and reflectivity spectra under biaxial and triaxial strains is systematically examined for *Pnma-1*, *Pnma-2*, and *P2*_1_/*m* phases. Such strain-dependent light-matter interactions play a crucial role in determining the performance of optoelectronic devices, including solar cells, photodetectors, and light-emitting diodes. Tables 4 and 5 summarize the static dielectric constant *ε*(0), the real and imaginary parts of the dielectric function (ε_1_(ω) and ε_2_(ω)), the absorption coefficient α­(ω), the refractive index *n*(ω), and the reflectivity *R*(ω) for −5%, 0%, and +5% biaxial and triaxial strain conditions.

**Table 5:** Static Dielectric Constant ε(0), Real Dielectric Constant ε_1_(ω), Imaginary Dielectric Constant ε_2_(ω), Absorption Coefficient α­(ω), Refractive Index *n*(ω), and Reflectivity *R*(ω) Values of the *Pnma-1*, *Pnma-2*, and *P2*_1_/*m* Materials under the Influence of −5%, 0%, and 5% Triaxial Strain

		*Pnma-1*			*Pnma-2*			*P2*_1_/*m*		
		polarization direction			polarization direction			polarization direction		
strain ratio	optic parameters	*x*	*y*	*z*	*x*	*y*	*z*	*x*	*y*	*z*
–5%	ε(0)	5.74	5.34	5.85	6.05	5.88	6.86	6.13	10.16	6.24
	ε_1_(ω)_max_	11.59	9.82	11.38	9.00	7.78	8.52	10.52	30.25	10.36
	ε_2_(ω)_max_	11.95	8.90	12.93	8.58	8.90	7.57	8.47	34.60	11.47
	α(ω)_max_	1.04 × 10^8^	1.16 × 10^8^	1.20 × 10^8^	1.10 × 10^8^	1.21 × 10^8^	1.04 × 10^8^	1.37 × 10^8^	1.56 × 10^8^	1.37 × 10^8^
	*n*(ω)_max_	3.52	3.24	3.48	3.11	2.82	3.03	3.32	5.62	3.31
	*R*(ω)%	79	75	77	69	74	62	65	71	63
0%	ε(0)	5.34	4.86	5.02	5.97	5.73	5.79	5.88	10.43	5.54
	ε_1_(ω)_max_	11.01	9.68	10.82	8.94	7.20	8.59	10.69	37.61	9.23
	ε_2_(ω)_max_	12.12	8.42	11.42	7.80	6.62	7.06	10.46	50.65	10.78
	α(ω)_max_	0.99 × 10^8^	1.04 × 10^8^	1.07 × 10^8^	0.91 × 10^8^	1.07 × 10^8^	0.90 × 10^8^	1.32 × 10^8^	1.47 × 10^8^	1.30 × 10^8^
	*n*(ω)_max_	3.46	3.24	3.39	3.06	2.76	3.00	3.43	6.56	3.18
	*R*(ω)%	77	70	77	63	65	63	58	82	61
5%	ε(0)	4.97	4.32	4.41	5.95	5.60	5.18	5.59	6.43	5.06
	ε_1_(ω)_max_	10.32	8.25	9.30	9.19	7.49	8.34	11.93	19.28	8.01
	ε_2_(ω)_max_	11.93	8.40	10.52	8.16	6.10	6.72	11.62	24.27	9.07
	α(ω)_max_	0.96 × 10^8^	0.94 × 10^8^	1.02 × 10^8^	0.84 × 10^8^	0.88 × 10^8^	0.82 × 10^8^	1.24 × 10^8^	1.34 × 10^8^	1.23 × 10^8^
	*n*(ω)_max_	3.41	2.97	3.12	3.11	2.79	2.95	3.54	4.81	2.93
	*R*(ω)%	74	57	77	60	52	61	52	62	55

Across all structures, the maximum values of *ε*_1_(ω) systematically decrease as the strain varies from −5% to +5%, confirming that increasing the tensile deformation weakens the polarization response. The *P2*_1_/*m* phase exhibits the highest polarizability in the visible range under y-polarized excitation, consistent with its large static dielectric constant observed at both −5% and 0% strain (Figure S3). Imaginary dielectric functions ε_2_(ω) of *Pnma-1*, *Pnma-2*, and *P2*_1_/*m* phases under the influence of triaxial strain and biaxial strain applied at −5%, 0%, and 5% rates are displayed in Figure S4.

The optical band gaps are calculated as 1.72, 1.95, and 2.02 eV for *Pnma-1*; 1.13, 1.20, and 1.35 eV for *Pnma-2*; and 1.95, 1.80, and 2.03 eV for *P2*_1_/*m* under −5%, 0%, and +5% triaxial strain, respectively. Under biaxial strain, band gap values slightly differ, yielding 1.88, 1.95, and 1.95 eV for *Pnma-1*; 1.13, 1.20, and 1.35 eV for *Pnma-2*; and 1.35, 1.80, and 2.10 eV for *P2*_1_/*m*. In all cases, absorption increases sharply from the optical band gap threshold and reaches a maximum in the ultraviolet region, indicating that photons across both visible and UV ranges are efficiently absorbed under biaxial/triaxial strain. Additionally, *Pnma-2* extends its absorption edge into the infrared region, suggesting its suitability for near-IR optoelectronic applications. For *Pnma-1*, the highest absorption coefficient occurs under −5% strain with z-polarized light, while for *Pnma-2* and *P2*_1_/*m*, the strongest response is calculated under y-polarized excitation at −5% strain (Figure S5). With increasing tensile strain, the maximum absorption values decrease slightly and shift toward lower photon energies, confirming that strain softens optical transitions. The overall absorption behavior under biaxial strain closely follows that of triaxial strain, although with marginally lower attenuation, indicating a comparable optical response at reduced energetic cost.

The strain-dependent refractive index results show a gradual decrease in both static and maximum *n*(ω) values for *Pnma-1* and *Pnma-2* as strain changes from −5% to +5%, while *P2*_1_/*m* retains a significantly larger index. Its peak refractive index of 6.56 at 0% strain under *y*-polarized excitation in the visible range suggests strong optical confinement and lower transparency compared to the orthorhombic phases (Figure S6). As shown in Figure S7, all structures exhibit high reflectance in the ultraviolet region, while reflectivity in the visible range remains moderate (20–40%). Increasing the tensile strain reduces the overall reflectance across the spectrum. The strong UV reflectance of all three phases points to their potential as UV-protective or reflective coatings, while the *P2*_1_/*m* phase with its high visible range reflectance, may serve as a candidate for wavelength-selective optical shielding or reflective components. Overall, such strain-dependent optical properties demonstrate that mechanical deformation provides an important route for tailoring light absorption, reflection, and confinement in KSnI_3_ perovskites, opening pathways toward polarization- and wavelength-engineered optoelectronic applications.

## 4 Conclusion

In conclusion, the structural, vibrational, electronic, optical, and elastic properties of orthorhombic (*Pnma-1* and *Pnma-2*), tetragonal (*P4/mmm* and *P4/mbm*), and monoclinic (*P2*_1_/*m*) KSnI_3_ perovskites were systematically investigated using density functional theory-based first-principles calculations. Phonon spectra revealed that the tetragonal phases were dynamically unstable. In contrast, the *Pnma-1*, *Pnma-2*, and *P2*_1_/*m* phases were found to be dynamically, thermally, and mechanically stable. From an electronic perspective, *Pnma-1* and *P2*_1_/*m* exhibited indirect semiconducting behavior, while *Pnma-2* and the tetragonal phases displayed direct band gaps, highlighting the role of crystal symmetry in governing the electronic structure. All stable phases exhibited strong polarization-dependent optical absorption in the visible-UV region with *Pnma-2* extending into the infrared, thereby enabling a broadband optical response. The *P2*_1_/*m* phase showed remarkable optical anisotropy, with a y-polarized absorption coefficient of 1.06 × 10^8^ cm^–1^ at 1.88 eV and reflectivity reaching 82% in the visible range. In addition, its high reflectivity suggests potential applications in reflective optical coatings or polarization-selective optical devices. Furthermore, biaxial and triaxial strain provided an effective means for tuning the band gap and optical response over a wide energy range without altering the semiconducting character, highlighting strain as a key parameter for tailoring optoelectronic properties. Overall, this work established KSnI_3_ as a tunable lead-free perovskite platform, where the interplay between symmetry and strain provides a robust framework for designing advanced optoelectronic materials.

## Supplementary Material



## Data Availability

No data was used for the research described in the article.

## Notes

The mechanical stability criteria for orthorhombic structures and monoclinic structures; strain-dependent evolution of external pressure, lattice parameters, and band gap in the orthorhombic *Pnma-1* phase under biaxial compressive and tensile strain (0–5%) (Table S1); strain-dependent evolution of external pressure, lattice parameters, and band gap in the orthorhombic *Pnma-1* phase under triaxial compressive and tensile strain (0–5%) (Table S2); strain-dependent evolution of external pressure, lattice parameters, and band gap in the orthorhombic *Pnma-2* phase under biaxial compressive and tensile strain (0–5%) (Table S3); strain-dependent evolution of external pressure, lattice parameters, and band gap in the orthorhombic *Pnma-2* phase under triaxial compressive and tensile strain (0–5%) (Table S4); strain-dependent evolution of external pressure, lattice parameters, and band gap in the monoclinic *P2*_1_/*m* phase under biaxial compressive and tensile strain (0–5%) (Table S5); strain-dependent evolution of external pressure, lattice parameters, and band gap in the monoclinic *P2*_1_/*m* phase under triaxial compressive and tensile strain (0–5%) (Table S6); calculated electronic band structures of orthorhombic *Pnma-2* phase under biaxial and triaxial strain ranging from −5% to 5% (Figure S1); calculated electronic band structures of monoclinic *P2*_1_/*m* phase under biaxial and triaxial strain ranging from −5% to 5% (Figure S2); real part of the dielectric function, ε_1_(ω), of the *Pnma-1*, *Pnma-2*, *and P2*_1_/*m* phases under triaxial and biaxial strain at −5%, 0%, and 5% (Figure S3); imaginary part of the dielectric function, ε_2_(ω), of the *Pnma-1*, *Pnma-2*, *and P2*_1_/*m* phases under triaxial and biaxial strain at −5%, 0%, and 5% (Figure S4); absorption coefficient, α­(ω), of the *Pnma-1*, *Pnma-2*, *and P2*_1_/*m* phases under triaxial and biaxial strain at −5%, 0%, and 5% (Figure S5); refractive index, *n*(ω), of the *Pnma-1*, *Pnma-2*, *and P2*_1_/*m* phases under triaxial and biaxial strain at −5%, 0%, and 5% (Figure S6); and reflectance ratio *R*(ω) of *Pnma-1*, *Pnma-2*, *P2*_1_/*m* materials under the influence of triaxial strain applied at −5%, 0% and 5% rates, biaxial strain applied at −5%, 0% and 5% rates (Figure S7) (PDF)
