# Supplementary material for: Strain-Tunable Electronic and Optical Properties of KSnI3 Perovskite Polymorphs: From Structural Stability to Optoelectronic Potential
Source: ACS Omega. 2026 May 2;11(18):27128–42. doi: 10.1021/acsomega.6c00784 (PMC13177007; doi:10.1021/acsomega.6c00784)
Supplement: Supplementary file 1 [file ao6c00784_si_001.pdf]

Supporting Information

Strain-Tunable Electronic and Optical Properties of $KSnI_3$ Perovskite Polymorphs: From Structural Stability to Optoelectronic Potential

Aynur Ayvalik[a], Kadir Can Dogan[b], Zebih Cetin[c], Mehmet Yagmurcukardes[c]* and Humeyra Orucu[d]*

[a] Department of Material Science and Engineering, The Graduate School of Natural and Applied Sciences, Ege University, 35100 Izmir, Turkey
[b] Department of Physics, Izmir Institute of Technology, 35430 Izmir, Turkey
[c] Department of Photonics, Izmir Institute of Technology, 35430 Izmir, Turkey
[d] Department of Physics, Ege University, 35100 Izmir, Turkey

** Corresponding authors: humeyra.orucu@ege.edu.tr and mehmetyagmurcukardes@iyte.edu.tr*

For orthorhombic structures, the mechanical stability criteria are given as follows:

$$C_{11} > 0; C_{22} > 0; C_{33} > 0; C_{44} > 0; C_{55} > 0; C_{66} > 0 \quad (1)$$

$$C_{11} + C_{22} + C_{33} + 2(C_{12} + C_{13} + C_{23}) > 0 \quad (2)$$

$$C_{11} + C_{22} - 2C_{12} > 0 \quad (3)$$

$$C_{22} + C_{33} - 2C_{23} > 0 \quad (4)$$

$$C_{11} + C_{33} - 2C_{13} > 0 \quad (5)$$

For monoclinic structures, the mechanical stability criteria can be written as follows:

$$C_{11} > 0; C_{22} > 0; C_{33} > 0; C_{44} > 0; C_{55} > 0; C_{66} > 0 \quad (6)$$

$$C_{11} + C_{22} + C_{33} + 2(C_{12} + C_{13} + C_{23}) > 0 \quad (7)$$

$$C_{22} + C_{33} - 2C_{23} > 0 \quad (8)$$

$$C_{44} + C_{66} - C_{46}^2 > 0 \tag{9}$$

$$C_{33} + C_{55} - C_{35}^2 > 0 \tag{10}$$

$$\left[C_{22}\left(C_{33}C_{55} - C_{35}^2\right) + 2C_{23}C_{25}C_{35} - C_{55}C_{23}^2 - C_{33}C_{25}^2\right] > 0 \tag{11}$$

$$g = C_{11}C_{22}\,C_{33} - C_{11}C_{23}^2 - C_{22}C_{13}^2 - C_{33}C_{12}^2 + 2(C_{12}C_{13}C_{23}) \tag{12}$$

$$\begin{aligned}\{2[C_{15}C_{25}(C_{33}C_{12} - C_{13}C_{23}) + C_{15}C_{35}(C_{22}C_{13} - C_{12}C_{23}) + C_{25}C_{35}(C_{11}C_{23} - C_{12}C_{13})] \\ - \left[C_{15}^2\left(C_{33}C_{22} - C_{23}^2\right) + C_{25}^2\left(C_{33}C_{11} - C_{13}^2\right) + C_{35}^2\left(C_{11}C_{22} - C_{12}^2\right)\right] + gC_{55}\} > 0\end{aligned} \tag{13}$$

Table S1. Strain-dependent evolution of external pressure, lattice parameters, and band gap in the orthorhombic *Pnma-1* phase under biaxial compressive and tensile strain (0-5%).

Compound	Applied Strain (%)	Biaxial Compressive Strain			Biaxial Tensile Strain		
		External pressure (GPa)	Lattice parameters (Å)	Band gap (eV)	External pressure (GPa)	Lattice parameters (Å)	Band gap (eV)
**$K_4Sn_4I_{12}$ / *Pnma-1***	0	0.000	a = 4.64<br>b = 10.12<br>c = 16.97	1.78	0.000	a = 4.64<br>b = 10.12<br>c = 16.97	1.78
	1	0.357	a =4.59<br>b =10.02<br>c =16.97	1.78	-0.303	a = 4.69<br>b = 10.23<br>c = 16.97	1.78
	2	0.741	a =4.55<br>b =9.92<br>c =16.97	1.78	-0.573	a = 4.73<br>b = 10.33<br>c = 16.97	1.78
	3	1.184	a =4.50<br>b =9.82<br>c =16.97	1.75	-0.808	a = 4.78<br>b = 10.43<br>c = 16.97	1.78
	4	1.673	a =4.45<br>b =9.72<br>c =16.97	1.75	-1.008	a =4.83<br>b = 10.53<br>c = 16.97	1.76
	5	2.133	a =4.41<br>b =9.62<br>c =16.97	1.61	-1.178	a = 4.87<br>b = 10.63<br>c = 16.97	1.75

Table S2. Strain-dependent evolution of external pressure, lattice parameters, and band gap in the orthorhombic *Pnma-1* phase under triaxial compressive and tensile strain (0-5%).

Compound	Applied Strain (%)	Triaxial Compressive Strain			Triaxial Tensile Strain		
		External pressure (GPa)	Lattice parameters (Å)	Band gap (eV)	External pressure (GPa)	Lattice parameters (Å)	Band gap (eV)
$K_4Sn_4I_{12}$ / *Pnma-1*	0	0.000	a=4.64<br>b=10.12<br>c=16.97	1.78	0.000	a=4.64<br>b=10.12<br>c=16.97	1.78
	1	0.551	a=4.59<br>b=10.02<br>c=16.80	1.75	-0.453	a=4.67<br>b=10.23<br>c=17.14	1.80
	2	1.200	a=4.55<br>b=9.92<br>c=16.70	1.72	-0.829	a=4.73<br>b=10.33<br>c=17.31	1.82
	3	1.966	a=4.50<br>b=9.82<br>c=16.46	1.68	-1.134	a=4.78<br>b=10.43<br>c=17.47	1.84
	4	2.853	a=4.45<br>b=9.72<br>c=16.29	1.63	-1.375	a=4.83<br>b=10.53<br>c=17.67	1.85
	5	3.868	a=4.41<br>b=9.62<br>c=16.12	1.56	-1.561	a=4.87<br>b=10.63<br>c=17.82	1.86

Table S3. Strain-dependent evolution of external pressure, lattice parameters, and band gap in the orthorhombic *Pnma-2* phase under biaxial compressive and tensile strain (0-5%).

Compound	Applied Strain (%)	Biaxial Compressive Strain			Biaxial Tensile Strain		
		External pressure (GPa)	Lattice parameters (Å)	Band gap (eV)	External pressure (GPa)	Lattice parameters (Å)	Band gap (eV)
$K_4Sn_4I_{12}$ / *Pnma-2*	0	0.000	a =8.81<br>b =7.90<br>c =11.83	0.82	0.000	a=8.81<br>b=7.90<br>c=11.83	0.82
	1	0.344	a =8.72<br>b =7.82<br>c =11.83	0.82	-0.306	a=8.89<br>b=7.98<br>c=11.83	0.82
	2	0.724	a =8.63<br>b =7.74<br>c =11.83	0.82	-0.578	a=8.98<br>b=8.06<br>c=11.83	0.81
	3	1.148	a =8.54<br>b =7.66<br>c =11.83	0.82	-0.831	a=9.07<br>b=8.14<br>c=11.83	0.81
	4	1.624	a =8.45<br>b =7.58<br>c =11.83	0.80	-1.053	a=9.16<br>b=8.22<br>c=11.83	0.81
	5	2.161	a =8.37<br>b =7.51<br>c =11.83	0.78	-1.258	a=9.25<br>b=8.30<br>c=11.83	0.81

Table S4. Strain-dependent evolution of external pressure, lattice parameters, and band gap in the orthorhombic *Pnma-2* phase under triaxial compressive and tensile strain (0-5%).

Compound	Applied Strain (%)	Triaxial Compressive Strain			Triaxial Tensile Strain		
		External pressure (GPa)	Lattice parameter (Å)	Band gap (eV)	External pressure (GPa)	Lattice parameter (Å)	Band gap (eV)
$K_4Sn_4I_{12}$ / *Pnma-2*	0	0.000	a=8.81<br>b=7.90<br>c=11.83	0.82	0.000	a=8,81<br>b=7,90<br>c=11,83	0.82
	1	0.616	a=8.72<br>b=7.82<br>c=11.71	0.82	-0.446	a=8,89<br>b=7,98<br>c=11,95	0.83
	2	1.108	a=8.63<br>b=7.74<br>c=11.59	0.82	-0.836	a=8,98<br>b=8,06<br>c=12,06	0.83
	3	1.792	a=8.54<br>b=7.66<br>c=11.47	0.81	-1.176	a=9,07<br>b=8,14<br>c=12,18	0.84
	4	2.584	a=8.45<br>b=7.58<br>c=11.35	0.80	-1.466	a=9,16<br>b=8,22<br>c=12,30	0.84
	5	3.512	a=8.37<br>b=7.51<br>c=11.24	0.79	-1.711	a=9,25<br>b=8,30<br>c=12,42	0.85

Table S5. Strain-dependent evolution of external pressure, lattice parameters, and band gap in the monoclinic *P2₁/m* phase under biaxial compressive and tensile strain (0-5%).

Compound	Applied Strain (%)	Biaxial Compressive Strain			Biaxial Tensile Strain		
		External pressure (GPa)	Lattice parameters (Å)	Band gap (eV)	External pressure (GPa)	Lattice parameters (Å)	Band gap (eV)
$K_2Sn_2I_6$ / *$P2_1/m$*	0	0.000	a=6.21<br>b=8.70<br>c=8.39	1.47	0.000	a=6.21<br>b=8.70<br>c=8.39	1.47
	1	0.338	a=6.15<br>b=8.61<br>c=8.39	1.50	-0.291	a=6.27<br>b=8.78<br>c=8.39	1.50
	2	0.726	a=6.08<br>b=8.52<br>c=8.39	1.52	-0.503	a=6.33<br>b=8.87<br>c=8.39	1.56
	3	1.152	a=6.02<br>b=8.43<br>c=8.39	1.56	-0.679	a=6.39<br>b=8.96<br>c=8.39	1.65
	4	1.620	a=5.96<br>b=8.35<br>c=8.39	1.61	-0.828	a=6.46<br>b=9.04<br>c=8.39	1.73
	5	2.132	a=5.90<br>b=8.26<br>c=8.39	1.66	-0.954	a=6.52<br>b=9.13<br>c=8.39	1.80

Table S6. Strain-dependent evolution of external pressure, lattice parameters, and band gap in the monoclinic *$P2_1/m$* phase under triaxial compressive and tensile strain (0-5%).

Compound	Applied Strain (%)	Triaxial Compressive Strain: External pressure (GPa)	Triaxial Compressive Strain: Lattice parameters (Å)	Triaxial Compressive Strain: Band gap (eV)	Triaxial Tensile Strain: External pressure (GPa)	Triaxial Tensile Strain: Lattice parameters (Å)	Triaxial Tensile Strain: Band gap (eV)
**$K_2Sn_2I_6$ / *$P2_1/m$***	0	0.000	a=6.21<br>b=8.7<br>c=8.39	1.47	0.000	a=6.21<br>b=8.70<br>c=8.39	1.47
	1	0.438	a=6.15<br>b=8.61<br>c=8.31	1.50	-0.374	a=6.27<br>b=8.78<br>c=8.48	1.49
	2	0.955	a=6.08<br>b=8.51<br>c=8.23	1.53	-0.637	a=6.33<br>b=8.87<br>c=8.56	1.57
	3	1.562	a=6.02<br>b=8.43<br>c=8.14	1.56	-0.850	a=6.39<br>b=8.96<br>c=8.64	1.65
	4	2.247	a=5.96<br>b=8.35<br>c=8.06	1.60	-1.018	a=6.46<br>b=9.04<br>c=8.73	1.73
	5	3.034	a=5.90<br>b=8.26<br>c=7.97	1.64	-1.149	a=6.52<br>b=9.13<br>c=8.81	1.81

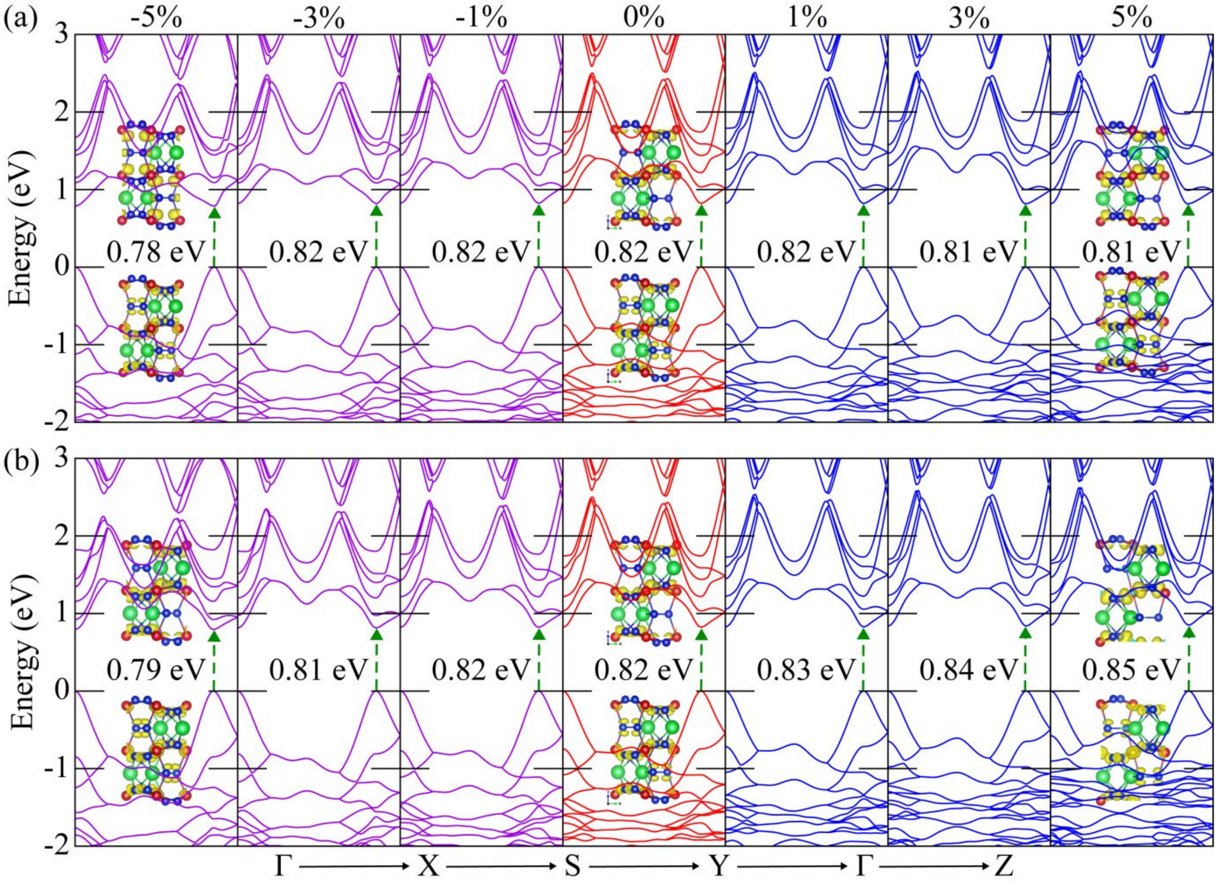

Figure S1. The calculated electronic band structures of orthorhombic *Pnma-2* phase under (a) biaxial and (b) triaxial strain ranging from -5% to 5%, along with the atomic orbital contributions to the VBM and CBM states at the strain limits. The isosurface value for the atomic orbitals is taken as 2 × 10^{-6} e/Å^3. K, Sn, and I atoms are represented in green, red, and blue, respectively.

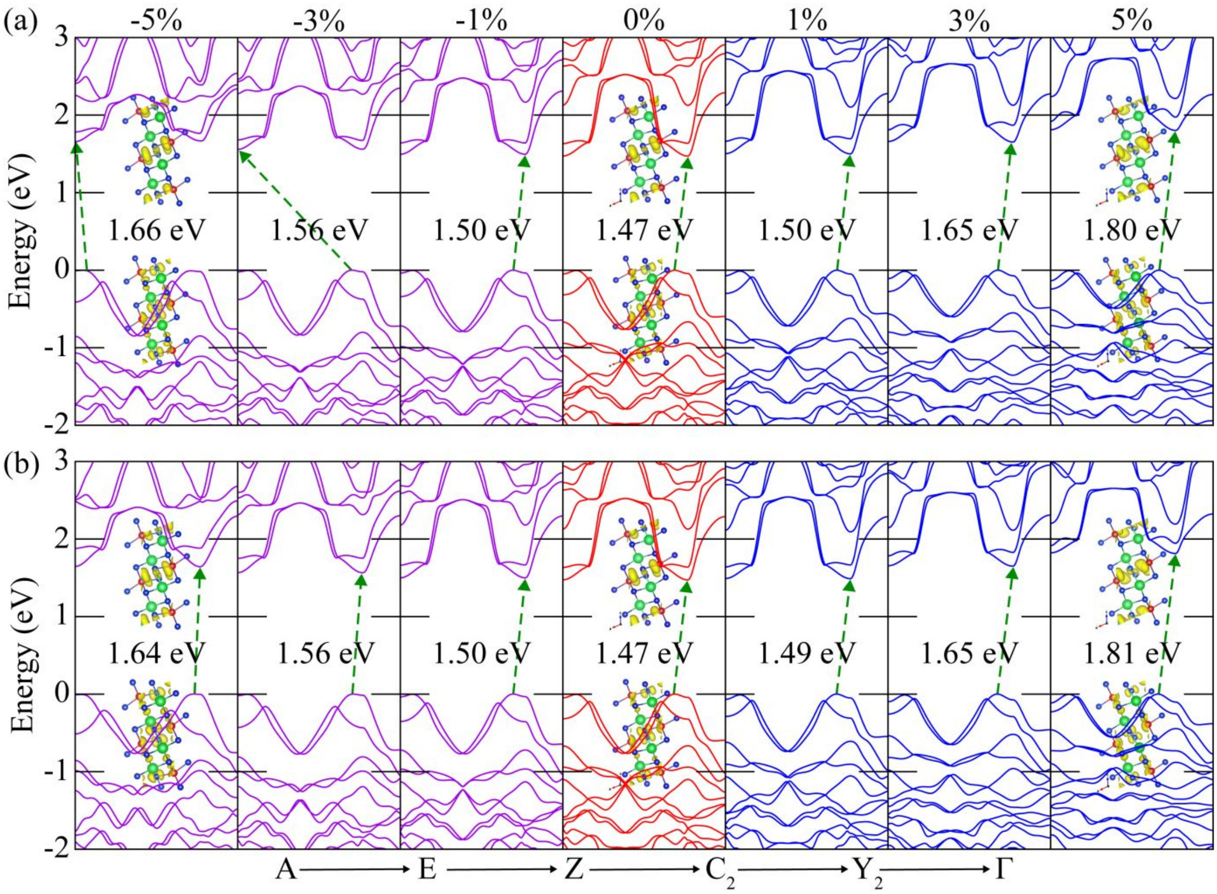

Figure S2. The calculated electronic band structures of monoclinic *P2₁/m* phase under (a) biaxial and (b) triaxial strain ranging from -5% to 5%, along with the atomic orbital contributions to the VBM and CBM states at the strain limits. The isosurface value for the atomic orbitals is taken as 3 × 10^{-6} e/Å^3. K, Sn, and I atoms are represented in green, red, and blue, respectively.

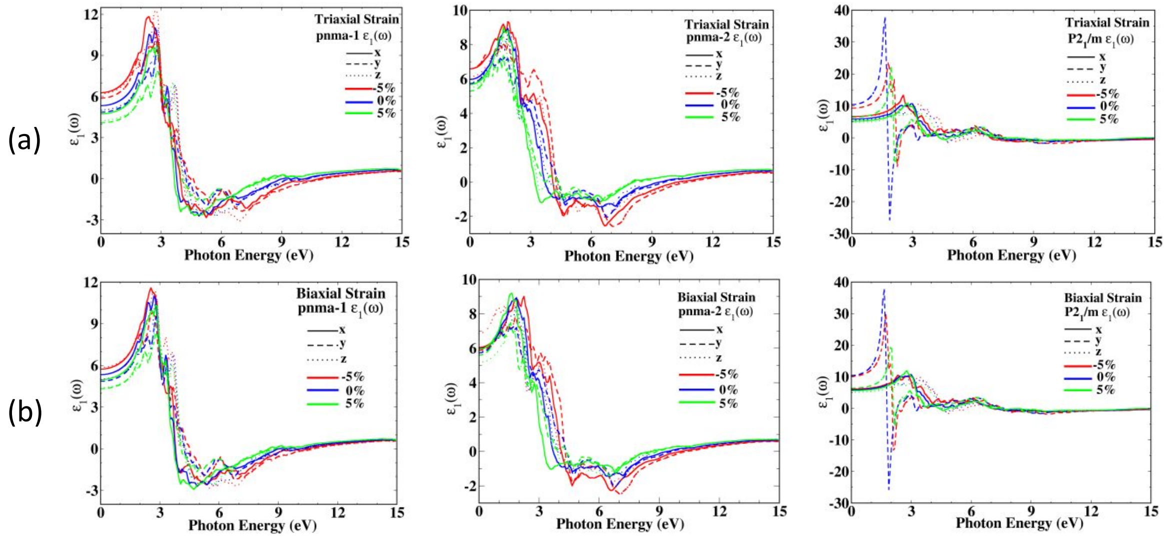

Figure S3. Real part of the dielectric function, $\varepsilon_1(\omega)$, of the *Pnma-1, Pnma-2, and P2₁/m* phases under (a) triaxial and (b) biaxial strain at -5%, 0%, and 5%.

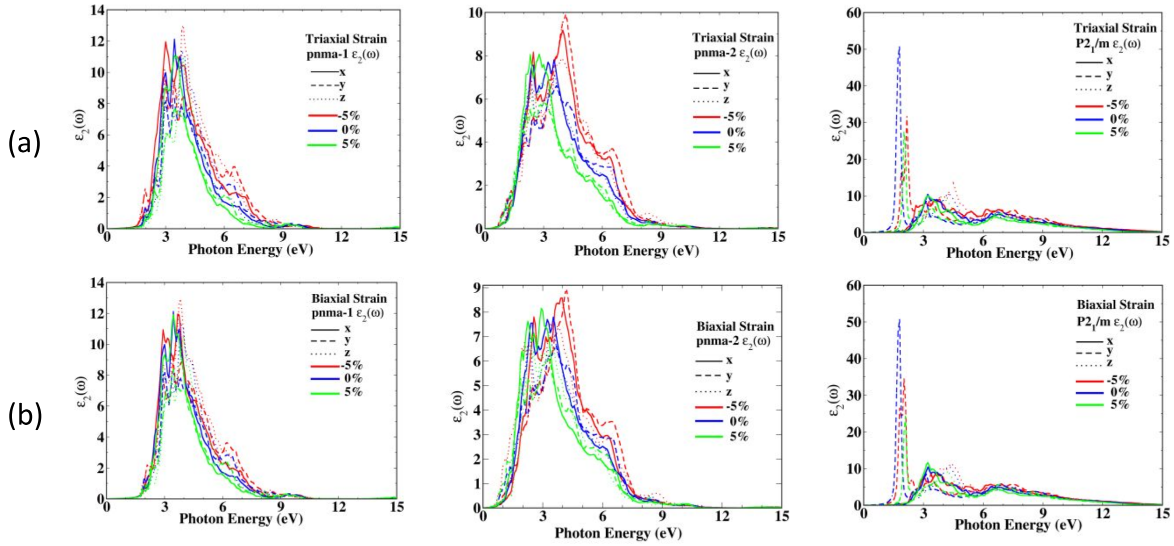

Figure S4. Imaginary part of the dielectric function, $\varepsilon_2(\omega)$, of the *Pnma-1, Pnma-2, and P2$_1$/m* phases under (a) triaxial and (b) biaxial strain at -5%, 0%, and 5%.

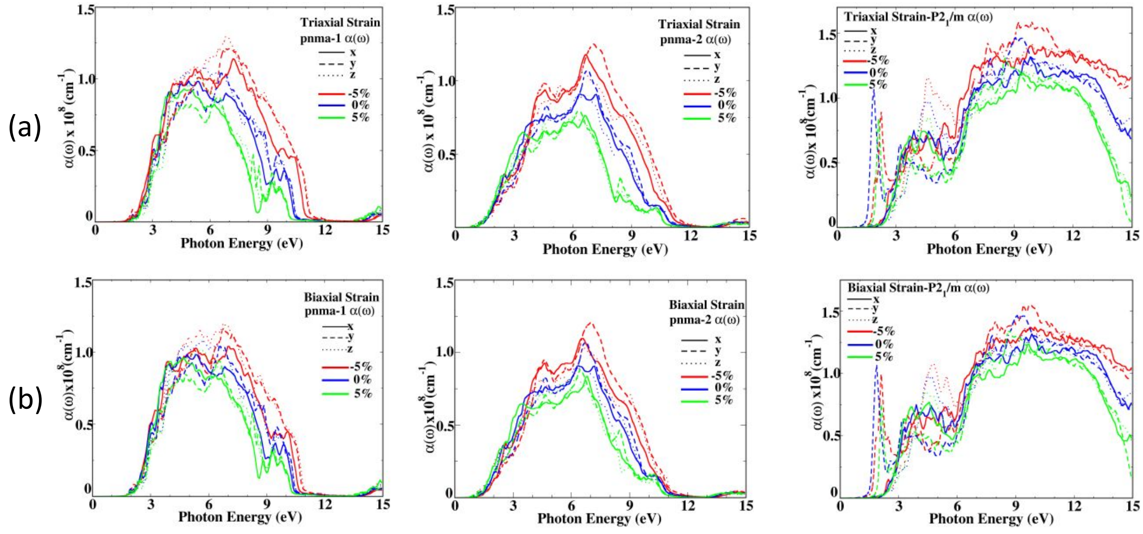

Figure S5. Absorption coefficient, $\alpha(\omega)$, of the *Pnma-1, Pnma-2, and P2$_1$/m* phases under (a) triaxial and (b) biaxial strain at -5%, 0%, and 5%.

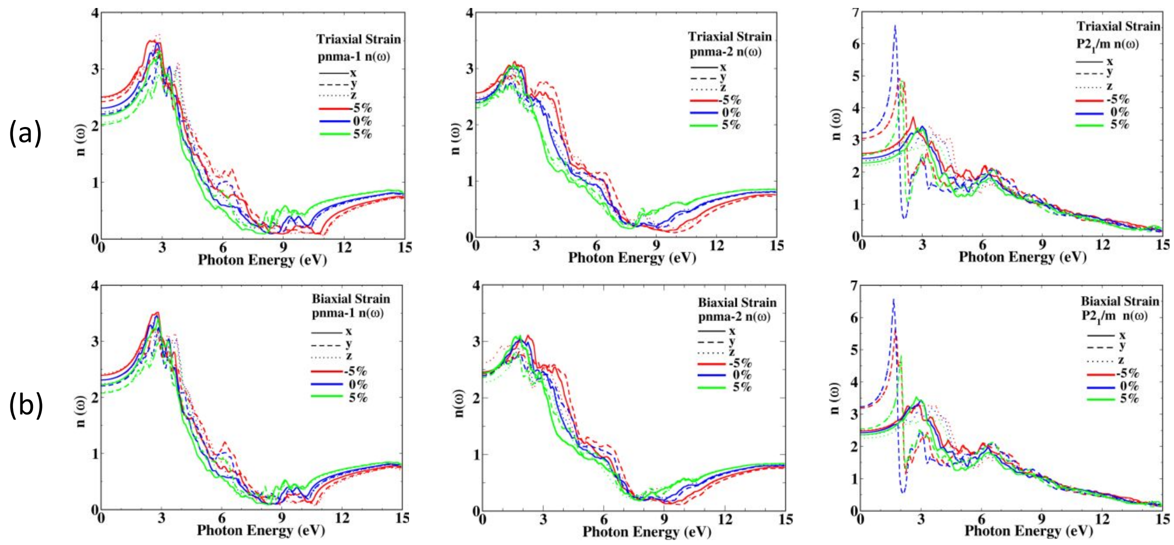

Figure S6. Refractive index, n(ω), of the *Pnma-1, Pnma-2, and P2$_1$/m* phases under (a) triaxial and (b) biaxial strain at -5%, 0%, and 5%.

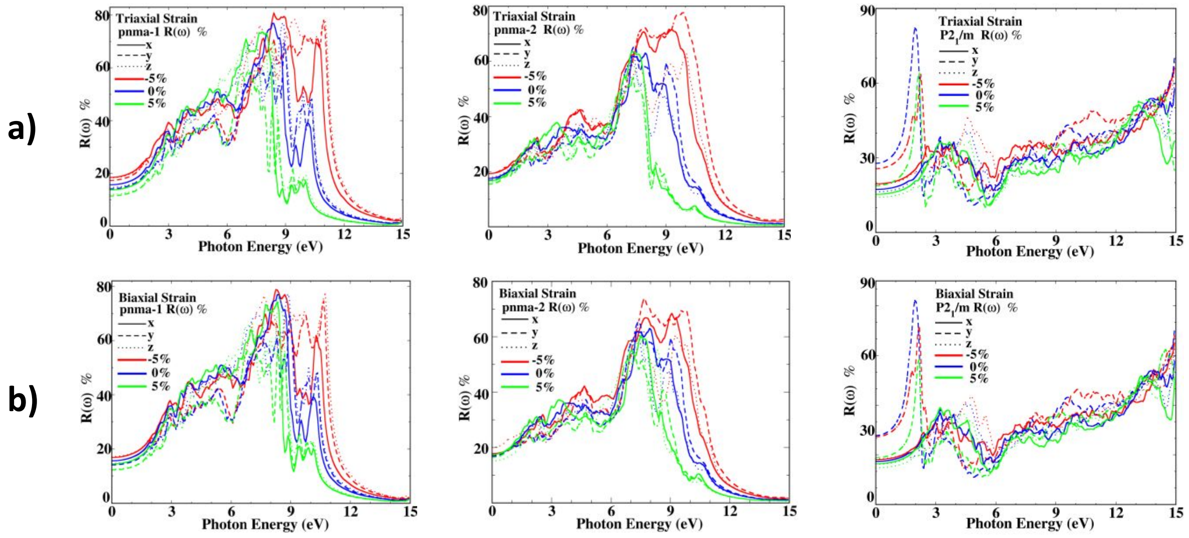

Figure S7. Reflectance ratio R(ω) of *Pnma-1, Pnma-2, P2$_1$/m* materials under the influence of a) triaxial strain applied at -5%, 0% and 5% rates, b) biaxial strain applied at -5%, 0% and 5% rates.
